# Why some minds bend, not break: an exploratory study of cognitive and psychological performance across levels of cognitive reserve in subjective cognitive decline

**DOI:** 10.3389/fpsyg.2026.1808150

**Published:** 2026-06-26

**Authors:** Annalisa Magnani, Antonia Pierobon, Valeria Torlaschi, Cira Fundarò, Marina Maffoni

**Affiliations:** 1 Department of Psychology, Catholic University of the Sacred Heart, Milan, Italy; 2 Psychology Unit, Montescano Institute, Istituti Clinici Scientifici Maugeri IRCCS, Montescano, Italy; 3 Neurophysiopathology Unit, Montescano Institute, Istituti Clinici Scientifici Maugeri IRCCS, Montescano, Italy

**Keywords:** cognitive rehabilitation, cognitive reserve, cognitive reserve index questionnaire, depressive symptomatology, psychological resilience, subjective cognitive decline

## Abstract

**Introduction:**

Subjective Cognitive Decline (SCD) is a heterogeneous at-risk condition along the Alzheimer's disease continuum, characterized by cognitive complaints despite preserved neuropsychological performance. Cognitive reserve (CR) may help explain individual differences in resilience to early cognitive and psychological vulnerability. This exploratory cross–sectional study examined whether global and domain-specific CR–education, occupational activity, and leisure time - were associated with differences in cognitive and psychological functioning in individuals with SCD.

**Methods:**

Fifty-one older adults with SCD underwent a comprehensive neuropsychological assessment and self-report measures of subjective complaints, depressive symptoms, and anxiety. Non-parametric analyses compared performance across low, medium, and high CR levels.

**Results:**

Cognitive performance was globally preserved, while depressive symptoms were clinically relevant in a substantial proportion of participants. Selective CR-related differences emerged: higher global CR was associated with better visuospatial-constructive performance; lower leisure-time CR was associated with faster performance on attentional and executive tasks; and occupational CR showed a non-monotonic association with depressive symptoms, with lower scores in the medium CR group. No differences emerged in other cognitive domains.

**Discussion:**

These findings support a multidimensional and domain-specific conceptualization of CR in SCD and highlight the clinical value of integrating CR into early cognitive aging assessment.

## 1 Introduction

Global increases in life expectancy are contributing to both population aging and the growing burden of age-related diseases. Neurocognitive disorders (NCDs) (American Psychiatric Association, 2022) are among the most pressing public health challenges (Pérez Palmer et al., 2022; Yu et al., 2023), imposing personal, social, and economic costs, emphasizing the urgency of understanding cognitive aging trajectories and developing strategies to preserve brain health across the lifespan (Sabayan et al., 2023). Alongside advances in treatment (Chen et al., 2022), progress depends on early detection and prevention strategies capable of intervening before decline becomes irreversible, helping to delay progression and reduce the burden on patients and caregivers (Mancini et al., 2025; Wang et al., 2020).

Given these considerations, Subjective Cognitive Decline (SCD) has emerged as an early marker of vulnerability (Maffoni and Magnani, 2025). It refers to self-perceived worsening of memory or other cognitive abilities despite normal performance on standardized neuropsychological tests (Jessen et al., 2014, 2020; Viviano and Damoiseaux, 2020). Epidemiological data suggest that SCD affects ~25% of individuals over age 60 (Röhr et al., 2020), with prevalence rates increasing in community-dwelling populations (Ribaldi et al., 2022). The Subjective Cognitive Decline Initiative (SCD-I), an international working group, proposed a conceptual framework defining SCD based on three fundamental criteria: (i) a self-experienced, persistent decline in cognitive functioning relative to a previously normal baseline; (ii) no evidence of an acute precipitating condition; and (iii) unimpaired performance on standardized cognitive tests, appropriately adjusted for age, sex, and education (Jessen et al., 2014). Moreover, as SCD can precede Mild Cognitive Impairment (MCI) and dementia, it is seen as a potential risk state along the Alzheimer's disease (AD) continuum (Jessen et al., 2020; Liew, 2020a; Numbers et al., 2021; van Harten et al., 2018).

The SCD-I expanded on this concept by including the SCD-Plus criteria – a set of features linked to a higher likelihood of underlying neurodegenerative processes and greater risk of progression to AD (Jessen et al., 2014; Kang et al., 2024; Wang et al., 2020). These features include subjective complaints specifically involving memory rather than other cognitive domains, associated concern about cognitive decline, the perception of performing worse than same-aged peers, informant confirmation of decline, onset of complaints within the previous 5 years and after the age of 60, as well as APOE ε4 carrier status and biomarker evidence consistent with AD pathology (Jessen et al., 2014; Zülke et al., 2026). Nevertheless, longitudinal evidence indicates that SCD is a heterogeneous condition, characterized by multiple possible trajectories rather than a uniform progression toward NCDs (Ribaldi et al., 2022): complaints may resolve when reversible causes—such as affective disorders or medication side effects—are addressed, or remain stable and reflect normal age-related state (Jessen et al., 2020; Liew, 2019, 2020a,b).

A growing body of research has identified several factors associated with SCD onset and progression. Non-modifiable risk factors—including older age, female sex, and genetic susceptibility—are consistently linked to higher prevalence of SCD and increased likelihood of progression to objective impairment (Li et al., 2023; Ma et al., 2024; Mazure and Swendsen, 2016; Mielke et al., 2014). Besides them there are other risk factors that can be modified (Istituto Superiore di Sanità., 2024). Lower educational attainment has been associated with a higher likelihood of SCD and subsequent progression to MCI; however, highly educated individuals may also report SCD due to greater awareness of subtle cognitive changes (Arora et al., 2024; Ma et al., 2024; Röhr et al., 2020). Similarly, psychiatric and psychosocial factors can play a central role (Amariglio, 2021; Sabatini et al., 2022): anxiety may amplify monitoring of cognitive performance, whereas depression has been linked to both brain changes and poorer cognition (Liew, 2019, 2020b; Ma et al., 2024; Sabatini et al., 2022; Si et al., 2020; Yates et al., 2017; Zlatar et al., 2018). Finally, lifestyle and vascular risk factors—including physical inactivity, obesity, smoking, hypertension, diabetes, and cardiovascular disease—further contribute to SCD prevalence and increase the risk of progression along the AD continuum (Livingston et al., 2020, 2024).

Although research on these risk factors is ongoing, growing evidence suggests that promoting healthy lifestyle patterns and reducing modifiable risk factors may represent a possible strategy for both the prevention of SCD and the mitigation of subsequent cognitive decline (Livingston et al., 2020, 2024). Against this backdrop, the heterogeneity of SCD can be framed within a resilience perspective (Stern et al., 2020, 2023). Although influential models conceptualize resilience in primarily neurocognitive terms—as an overarching construct subsuming cognitive reserve, brain maintenance and brain reserve (Stern et al., 2020, 2023) – the prominence of affective symptoms in SCD suggests that psychological resources should be considered alongside cognitive ones, as partly distinct yet complementary contributors to whether and how vulnerability is expressed as subjective decline (Arola et al., 2021; Maffoni et al., 2025b). Within this framework, cognitive reserve represents the cognitive component: CR is the brain's ability to sustain cognitive function better than expected given the degree of brain change by adaptively recruiting neural networks and adopting alternative cognitive strategies (Stern et al., 2023). Rather than preventing neural damage, it modulates how pathology translates into clinical symptoms (Gamble et al., 2025; Jia et al., 2021; McKenzie et al., 2023). Consequently, higher levels of CR have been linked to delayed onset of neurodegenerative conditions and better cognitive functioning in normal aging, with important clinical and diagnostic implications, particularly in early and preclinical stages (Stern et al., 2020). Indeed, high CR may mask early deficits—allowing individuals to tolerate greater neuropathology before measurable impairment—thereby complicating early identification and potentially leading to a sharper decline once symptoms emerge (Devita et al., 2020). Therefore, incorporating CR assessment into clinical evaluation may support earlier detection of subtle changes, improve interpretation of cognitive test results, and inform personalized intervention strategies (Devita et al., 2020; Gamble et al., 2025; Marmor et al., 2025). Traditionally, years of education have been used as a proxy for CR. However, education captures only part of lifetime cognitive enrichment. Therefore, CR is increasingly seen as a multidimensional, encompassing both static proxies—such as educational attainment—and dynamic proxies—including engagement in leisure activities, occupational complexity, and physical activity—which may differentially contribute to cognitive resilience across the lifespan (Ferrari-Díaz et al., 2025; Malek-Ahmadi et al., 2017; McGarrigle et al., 2019). The Cognitive Reserve Index questionnaire (CRIq) (Nucci et al., 2012) was developed to operationalize this multidimensional perspective, integrating educational attainment, occupational history and complexity, as well as leisure activities across the lifespan (Nucci et al., 2012; Mondini et al., 2023).

However, despite the availability of such multidimensional instruments, many studies continue to operationalize CR as a single composite index, often relying on education or a simple aggregate score. Yet, these approaches may fail to capture the distinct and domain-specific contributions of its static and dynamic components (Ferrari-Díaz et al., 2025; Mazzeo et al., 2019). This limitation is particularly relevant in SCD, where educational, lifestyle, and psychosocial factors may differentially relate to specific cognitive processes and subjective experiences. Hence, adopting a multidimensional perspective on CR may help to explain functional variability within SCD and capture early cognitive and psychological differences that that are not adequately explained by global reserve indices alone (Ferrari-Díaz et al., 2025). Thus, the present study aimed to investigate whether different levels of CR—operationalized using the short version of the CRIq questionnaire (Mondini et al., 2023), both as a global index and across its subdomains (education, working activity, and leisure time)—are associated with differences in cognitive and psychological functioning in individuals with SCD. By examining these associations from a non-assumptive, component-specific perspective, the study seeks to contribute to a more refined understanding of early cognitive and affective aging in SCD.

## 2 Materials and methods

### 2.1 Study framework and design

This study examines CR—and its components—in relation to cognitive and psychological functioning in individuals with SCD, using cross-sectional baseline data from the MASCoD project (Multidimensional Assessment of Subjective Cognitive Decline). MASCoD is a prospective, controlled clinical trial aimed at validating a new multidimensional screening tool for SCD. The form and its preliminary validation have been discussed in other publications (Maffoni et al., 2022, 2025a), and the full study protocol is pre-registered on ClinicalTrials.gov (Identifier: NCT05815329) (ClinicalTrials.gov, n.d.). Briefly, upon enrollment, participants undergo a standardized baseline assessment that includes a neurological examination, thorough neuropsychological and psychological evaluations, and brain ^18^F-FDG PET imaging where clinically indicated. While the core project also includes a 2-month technology-driven cognitive training intervention, the present study focuses exclusively on cross-sectional baseline data.

### 2.2 Eligibility, enrollment, and procedures

From July 2022 potential participants were consecutively recruited from the Neurophysiopathology Unit of the IRCCS ICS Maugeri Institute (Montescano, Italy), included in the Centers for Cognitive Disorders and Dementias (CCDDs) (Bacigalupo et al., 2024) within the Italian national network. These centers adhere to standardized Diagnostic Therapeutic Care Pathways (DTCPs) that ensure uniform diagnostic work-up, testing, rehabilitation, and follow-up care consistent with international guidelines for cognitive disorders (Frisoni et al., 2024; NICE, 2018).

During routine neurological consultations clinicians identified individuals presenting with subjective cognitive complaints and, after preliminary evaluation, flagged those eligible for further assessment within the MASCoD protocol, in accordance with the conceptual framework for SCD proposed by the SCD-I (Jessen et al., 2014). The inclusion criteria considered were the following: (i) self-reported SCD without a prior diagnosis of cognitive or neurological disorders, (ii) completion of formal education within the Italian school system, (iii) patients aged 55 years or older, (iv) adequate comprehension of study procedures, and (v) voluntary participation without financial compensation. Instead, patients were prevented to take part to the study if presenting any of the following exclusion criteria: (i) severe or unstable medical conditions (e.g., severe cardiac or respiratory disease, active neoplasia); (ii) a current or past psychiatric diagnosis according to DSM-5-TR (American Psychiatric Association, 2022), (iii) previously diagnosed mild cognitive impairment or dementia, (iv) clinically relevant visual-perceptual or hearing deficits, (v) illiteracy, and (vi) refusal or inability to participate.

Eligible participants provided written informed consent before undertaking the baseline visit—which included MASCoD screening procedure, a comprehensive neuropsychological battery, and neuroimaging examinations when clinically indicated. Participants were informed of their right to withdraw at any time without affecting their clinical care, and no financial compensation was provided. The study was conducted in accordance with the Declaration of Helsinki (World Medical Association, 2013) and was approved by the ICS Maugeri Ethics Committee (Protocol CE 2666, 26 July 2022).

### 2.3 Measurements

Given that SCD status had been established clinically prior to enrollment, two self-report instruments were administrated to quantify the severity of subjective cognitive complaints. The first is the self-report Cognitive Functional Index (CFI; Chipi et al., 2018), which yields a total score from 0 to 14. Mean scores are of 3.30 ± 2.44. Therefore, higher scores reflect greater self-perceived cognitive difficulties. The second is the Multidimensional Assessment of Subjective Cognitive Decline (MASCoD; Maffoni et al., 2025a), a screening tool yielding a total score from 0 to 21, where higher scores represent more self-perceived cognitive difficulties. Instead, CR was assessed using the short form of the Cognitive Reserve Index Questionnaire (s-CRIq; Mondini et al., 2023). This instrument evaluates three macro-domains—education, occupational activity, and leisure activities—and provides four indices: CRI-Education, CRI-WorkingActivity, CRI-LeisureTime, and a global CRI. The scores are grouped into five different levels: low (< 70 points), medium-low (71-−84 points), medium (85–114 points), medium-high (115–130 points), and high (> 130 points).

In parallel, cognitive functioning was evaluated using a comprehensive neuropsychological battery administered in accordance with DTCP and CCDD clinical guidelines (Frisoni et al., 2024; NICE, 2018). The battery was designed to provide a broad characterization of global cognition and domain-specific abilities, including attention, executive functions, short-term and working memory, long-term memory, language, visuospatial abilities, and constructive praxis. For all instruments, raw scores were corrected for age and education based on available Italian normative data, and these adjusted scores were used in subsequent analyses. Given the number of tests administered, a concise description of each measure is provided and summarized in Table 1, with instruments grouped by cognitive domain as considered in the analyses.

**Table 1:** Overview of the neuropsychological measures used for the cognitive assessment.

Assessed cognitive domains	Neuropsychological tests	Assessed cognitive functions	Scores range, interpretation and normative values^*^
Global cognition	Mini Mental State Examination (MMSE; Foderaro et al., 2022)	Global cognitive functioning	Scores range from 0 to 30, with higher scores indicating better performance. Adjusted scores ≥ 26.02 fall within the normal range
	Addenbrooke's Cognitive Examination III (ACE-III; Pigliautile et al., 2019)	Global cognitive functioning	Scores range from 0 to 100, with higher scores indicating better performance. Adjusted scores ≥ 68.68 fall within the normal range
Attention and executive functions	Frontal assessment battery (FAB; Aiello et al., 2022)	Main frontal executive functions	Scores range from 0 to 18, with higher scores indicating better performance. Adjusted scores ≥ 12.02 fall within the normal range
	Trail making test A (TMT-A; Siciliano et al., 2019)	Selective attention and visuospatial search	Score corresponds to completion time, with faster completion time indicative of a better performance. Adjusted scores ≤ 127 s fall within the normal range
	Trail making test B (TMT-B; Siciliano et al., 2019)	Alternating attention	Score corresponds to completion time, with faster completion time indicative of a better performance. Adjusted scores ≤ 294 s fall within the normal
	Stroop test time effect (Caffarra et al., 2002b)	Inhibition and sensitivity to interference	Score corresponds to completion time, with faster completion time indicative of a better performance. Adjusted scores ≤ 36.91 s fall within the normal range
	Stroop test errors effect (Caffarra et al., 2002b)	Inhibition and sensitivity to interference	Score corresponds to the number of errors committed, with less error indicative of a better performance. Adjusted scores ≤ 4.23 fall within the normal range
Language	Phonemic fluency test (FAS; Costa et al., 2014),	Phonemic fluency	Score corresponds to the number of words produced, with higher scores indicative of a better performance. Adjusted scores ≥ 17.77 fall within the normal range
	Semantic fluency test (Colors—Animals—Fruits; Costa et al., 2014)	Semantic fluency	Score corresponds to the number of words produced, with higher scores indicative of a better performance. Adjusted scores ≥ 28.34 fall within the normal range
Short-term memory	Digit span forward (DSF; Monaco et al., 2013)	Verbal short-term memory	Scores range from 0 to 9, with higher scores indicating better performance. Adjusted scores ≥ 4.26 fall within the normal range
	Cubes' Span Forward (CSF; Monaco et al., 2013)	Visuospatial short-term memory	Scores range from 0 to 9, with higher scores indicating better performance. Adjusted scores ≥ 3.46 fall within the normal range
Working memory	Digit span backward (DSB; Monaco et al., 2013)	Verbal working memory	Scores range from 0 to 8, with higher scores indicating better performance. Adjusted scores ≥ 2.65 fall within the normal range
	Cubes' span backward (CSB; Monaco et al., 2013)	Visuo-spatial working memory	Scores range from 0 to 8, with higher scores indicating better performance. Adjusted scores ≥ 3.17 fall within the normal range
Long-term memory	Rey–Osterrieth Complex Figure – Recall (ROCF Recall; Caffarra et al., 2002a)	Visuospatial long-term memory	Scores range from 0 to 36, with higher scores indicating better performance. Adjusted scores ≥ 9.47 fall within the normal range
	Rey's auditory verbal learning test—immediate recall (RAVLT—immediate recall; Carlesimo et al., 1996)	Verbal learning abilities	Scores range from 0 to 75, with higher scores indicating better performance. Adjusted scores ≥ 28.53 fall within the normal range
	Rey's auditory verbal learning test—delayed recall (RAVLT – delayed recall; Carlesimo et al., 1996)	Verbal long-term memory	Scores range from 0 to 15, with higher scores indicating better performance. Adjusted scores ≥ 4.69 fall within the normal range
Constructive praxis and visuospatial abilities	Rey–Osterrieth complex figure—Copy (ROCF Copy; Caffarra et al., 2002a)	Visuo-constructive and visuospatial abilities	Scores range from 0 to 36, with higher scores indicating better performance. Adjusted scores ≥ 28.88 fall within the normal range
	Clock drawing test (CDT; Caffarra et al., 2011)	Visual-spatial and planning skills	Scores range from 0 to 61, with higher scores indicating better performance. Adjusted scores ≥ 42.17 fall within the normal range

^*^Normative values are referred to Italian normative population.

Finally, psychological functioning was evaluated using self-report questionnaires. The Patient Health Questionnaire-9 (PHQ-9; Spitzer et al., 1999; Kroenke et al., 2001) was used to measure depressive symptoms over the previous 2 weeks. Scores range from 0 to 27, with higher scores indicating greater depressive symptom burden. Clinical cut-offs are ≥ 5 for at least mild depressive symptoms (0–4 none, 5–9 mild, 10–14 moderate, 15–19 moderately severe, ≥ 20 severe). Anxiety symptoms over the previous 2 weeks were assessed with the Generalized Anxiety Disorder-7 (GAD-7; Spitzer et al., 2006; Kroenke et al., 2007). Similarly to the previous test, clinical cut-offs are ≥ 5 for at least mild anxiety symptoms (0–4 none, 5–9 mild, 10–14 moderate, ≥ 15–19 severe).

### 2.4 Data analysis

Descriptive statistics were computed for all study variables. Categorical and ordinal variables were summarized using frequencies and percentages, while continuous variables were reported as means and standard deviations. Distributional assumptions were assessed using Shapiro-Wilk tests and graphical diagnostics. Given the frequent deviations from normality observed in this sample and the limited size of the cohort, non-parametric statistical methods were adopted for group comparisons. Consistent results across methods led to reporting only Shapiro-Wilk values in the main tables, with full diagnostics in Supplementary material.

One-sample *t*-tests were used to compare cognitive scores with established normative cut-offs and to determine whether psychological measures exceeded clinical thresholds (Maffoni et al., 2025a). For the s-CRIq, standard normative categories yielded marked asymmetry and numerically imbalanced groups. To enable balanced between-group comparisons in this specific sample while preserving the study's original design, total and subdomain CRIq scores (Education, Working Activity, Leisure Time) were recoded into three categories (low, medium, high) based on sample tertiles.

Prior to outcome analyses, equivalence across CR groups with respect to potential confounding variables (e.g., age, sex, and relevant clinical or vascular risk factors)—selected for their established association with SCD and cognitive aging (Hao et al., 2026; Zülke et al., 2026)—was evaluated using Kruskal-Wallis tests followed by Dwass-Steel-Critchlow-Fligner (DSCF) *post-hoc* pairwise comparisons where significant effects emerged (*p* < 0.05) (see Supplementary material). Variables inherently embedded in the computation of the s-CRIq (e.g., years of education, occupational history, housing status) were not tested as confounders, as they directly contribute to index construction. Additionally, to examine the relationship between CR and subjective complaint severity, Spearman correlations were computed between continuous CRI scores—total and subdomain—and CFI and MASCoD scores as outcomes.

Differences in cognitive and psychological performance across CR levels (low, medium, high) were examined separately for the CRI total and each CRI subdomain. Given the non-normal distributions, Kruskal-Wallis tests were used. As described in the previous section, outcome measures were organized into conceptual cognitive domains (i.e., global cognition, attention and executive functions, language, short-term memory, working memory, long-term memory, constructive praxis and visuospatial abilities). To control for inflation of Type I error due to multiple omnibus tests within each domain, Benjamini-Hochberg false discovery rate (FDR) correction was applied at the domain level (Benjamini and Hochberg, 1995). Only omnibus effects that survived FDR correction were considered statistically meaningful and were followed up with *post-hoc* analyses with DSCF—non-parametric multiple comparison method that intrinsically controls the family-wise error rate across all pairwise contrasts (Douglas and Michael, 1991).

Effect sizes for Kruskal-Wallis tests were quantified using epsilon-squared (ε^2^), with conventional benchmarks for interpretation: small (>0.01), medium (>0.06), and large (>0.14) (Cohen, 1988).

## 3 Results

### 3.1 Sociodemographic profile

A total of 51 individuals were enrolled. Their baseline sociodemographic and clinical characteristics are summarized in Table 2. The sample consisted primarily of older adults (69.73 ± 9.32) and was mostly female (70.6%). Educational attainment was generally intermediate (10.31 ± 3.89). Most participants were married or cohabiting (64.7%), and the majority were retired (68.6%).

Table 2Sociodemographic, clinical and lifestyle characteristics of the study sample.

**Table:** 

Sociodemographic variables					
Variable	*N*	Missing	M ±SD	Shapiro-Wilk	
				**W**	***p***
Age	51	0	69.73 ± 9.32	0.954	0.048
Years of schooling	51	0	10.31 ± 3.89	0.927	0.004
Weight (kg)	43	8	70.72 ± 13.64	0.963	0.179
Height (m)	43	8	1.64 ± 0.08	0.944	0.036
BMI	43	8	26.32 ± 5.15	0.905	0.002

**Table:** 

Variable	Levels	*N*	% of Total
Gender	Female	36	70.6%
	Male	15	29.4%
Marital status	Single	2	3.9%
	Married/Cohabiting	33	64.7%
	Widowed	11	21.6%
	Separated/Divorced	5	9.8%
Study Title	Elementary school graduation	10	19.6%
	Junior high school	17	33.3%
	High school graduation	19	37.3%
	Bachelor's degree	0	0.0%
	Master's degree	3	5.9%
	Postgraduate specialization	2	3.9%
Employment status	Self-employed	3	5.9%
	Full-time employee	5	9.8%
	Part-time employee	4	7.8%
	Homemaker	2	3.9%
	Unemployed	2	3.9%
	Retired	35	68.6%
Socio-family support	Spouse/Partner	28	54.9%
	Son/Daughter	19	37.3%
	Parent	0	0.0%
	Other Family Member	2	3.9%
	Caretaker	0	0.0%
	Other No Family Member	0	0.0%
	Nobody	2	3.9%
Clinical & lifestyle variables			
Physical activity	Yes	24	47.1%
	No	22	43.1%
	No response	5	9.8%
Overweight	Yes	25	49.0%
	No	26	51.0%
Alcohol	Yes	11	21.6%
	No	38	74.5%
	No response	2	3.9%
Smoking	Yes	8	15.7%
	No	25	49.0%
	Former smoker	15	29.4%
	No response	3	5.9%
Psychoactive drug addiction	Yes	0	0.0%
	No	49	96.1%
	No response	2	3.9%
Family history of NCDs	Yes	22	43.1%
	No	24	47.1%
	No response	5	9.8%
Diabetes	Yes	9	17.7%
	No	40	78.4%
	No response	2	3.9%
Hyperuricemia	Yes	0	0%
	No	49	96.1%
	No response	2	3.9%
Hypertension	Yes	24	47.1%
	No	25	49.0%
	No response	2	3.9%
Dyslipidemia	Yes	10	19.6%
	No	39	76.5%
	No response	2	3.9%

M, mean; N, number of participants; SD, standard deviation.

From a clinical and lifestyle standpoint, nearly half reported engaging in physical activity (47.1%). Overweight status was common (49%), consistent with a mean BMI of 26.32 ± 5.12 among those with available data. Hypertension was frequent (47.1%), whereas dyslipidemia affected roughly one fifth of the sample (19.6%). Diabetes was reported by 17.7% of participants. Smoking rates were low, but nearly one third identified as former smokers. Alcohol use was uncommon. Finally, 43.1% reported a family history of non-communicable diseases.

### 3.2 Neuropsychological and psychological performances

As shown in Table 3, participants performed well above the clinical cut-offs across all neuropsychological measures. One-sample *t*-tests confirmed large and highly significant deviations from impairment thresholds across every cognitive domain, indicating preserved cognitive functioning consistent with the expected SCD profile.

**Table 3:** Neuropsychological and psychological profile of the participants.

Variable	*N*	Missing	M ±SD	Shapiro-Wilk		Normative	t (*p*)
				W	*p*		
Neuropsychological tests							
MMSE	51	0	28.98 ± 1.26	0.806	< 0.001	≥ 26.02	−16.74^***^
ACE-III	44	7	87.68 ± 7.56	0.974	0.416	≥ 68.68	−16.16^***^
FAB	51	0	15.12 ± 2.23	0.961	0.089	≥ 12.02	−9.96^***^
Phonemic fluency	51	0	35.52 ± 9.05	0.986	0.815	≥ 17.77	−4.60^***^
Semantic fluency	50	1	46.85 ± 10.17	0.929	0.005	≥ 28.34	−12.96^***^
TMT A	51	0	33.35 ± 16.48	0.982	0.635	≤ 127.0	40.59^***^
TMT B	50	1	91.64 ± 50.93	0.930	0.006	≤ 294.0	28.09^***^
Stroop Time	49	2	16.87 ± 7.90	0.942	0.017	≤ 36.91	17.75^***^
Stroop Errors	49	2	0.81 ± 2.31	0.410	< 0.001	≤ 4.23	10.35^***^
DSF	51	0	5.61 ± 0.92	0.971	0.240	≥ 4.26	−10.50^***^
DSB	51	0	4.25 ± 1.05	0.873	< 0.001	≥ 2.65	−10.88^***^
CSF	51	0	5.43 ± 1.33	0.925	0.003	≥ 3.46	−10.57^***^
CSB	51	0	4.72 ± 1.48	0.914	0.001	≥ 3.17	−7.47^***^
RAVLT Immediate Recall	51	0	45.21 ± 9.46	0.905	0.296	≥ 28.53	−12.59^***^
RAVLT Delayed Recall	51	0	9.92 ± 3.82	0.961	0.096	≥ 4.69	−9.78^***^
ROCF Recall	51	0	16.06 ± 6.84	0.989	0.929	≥ 9.47	−6.88^***^
ROCF Copy	51	0	31.61 ± 5.16	0.796	< 0.001	≥ 28.88	−4.25^***^
CDT	51	0	56.29 ± 7.63	0.617	< 0.001	≥ 42.17	−13.22^***^
Psychological tests							
CFI	41	10	4.70 ± 2.71	0.961	0.170	≥ 5.74	2.47^*^
MASCoD	49	2	11.14 ± 3.56	0.923	0.211	-	-
PHQ-9	51	0	7.19 ± 5.23	0.923	0.003	≥ 5.0	−2.94^**^
GAD-7	51	0	6.10 ± 4.33	0.882	< 0.001	≥ 5.0	−1.81

^*^p < 0.05.

^**^ *p* < 0.01.

^***^ *p* < 0.001.

M, mean; N, number of participants; SD, standard deviation; *t*, Student's *t*-distribution; p, probability value (significance level).

With respect to subjective cognitive complaints, mean CFI scores were significantly below the clinical cut-off, indicating that, at the group level, self-reported cognitive concerns did not reach clinically significant severity despite the presence of a subjective complaint required for inclusion.

MASCoD scores were likewise assessed. Although no established clinical cut-off is currently available for this instrument, prior studies and clinical use indicate that higher scores are associated with an increased risk of subsequent objective cognitive decline (Gabbatore et al., 2025; Maffoni et al., 2025a). In the present sample, the MASCoD score was slightly above the distribution average and showed a moderate positive correlation with CFI scores (*r* = 0.461, *p* = 0.003). This finding supports the presence of overall modest subjective cognitive difficulties in this cohort.

In contrast, depressive symptoms were above the clinical threshold—suggesting at least mild depressive symptomatology—whereas anxiety levels did not differ significantly from the cut-off.

### 3.3 Sociodemographic profile

Standard s-CRIq normative categories (Mondini et al., 2023) indicated an overall medium-high CR profile but yielded markedly skewed distributions, with a clear floor effect in the lower categories and a pronounced clustering in the medium range. CRI total scores (M = 105, SD = 16.4) were predominantly classified as medium (68.9%), with few participants at the extremes. CRI education scores (M = 103, SD = 11.2) were even more homogeneous, with 80.4% of the sample in the medium category and no cases in the low or high categories, reflecting the limited variability. A similar even though less pronounced pattern emerged for CRI working activity and leisure time. Indeed, CRI working activity (M = 101, SD = 19.5) has half of the patient in the middle category (52.9%) and nearly the other half in the medium-low and high levels. CRI leisure time (M = 108, SD = 24.7) is the only subindex with the largest spread, with participants represented across the full range from low to high.

Given that the use of standard s-CRIq categories in this small and monocentric sample produced highly unbalanced and skewed distributions—particularly the absence of low-CR cases and the excessive concentration in the medium category – CRI scores were recoded into three groups based on sample tertiles (low, medium, high; Table 4), leading to a more balanced group size across all s-CRIq indices.

**Table 4:** Cognitive reserve.

Variable	*N*	M ±SD	Percentiles		Shapiro-Wilk		Levels according to s-CRIq		Levels according to tertiles	
			33.33^rd^	66.67^th^	W	*p*	Levels	*N* (%)	Levels	*N* (%)
CRI total	51	105 ± 16.4	95.0	111	0.955	0.051	Low	0 (0%)	Low	15 (29.4%)
							Medium-low	2 (3.9%)	Medium	18 (35.3%)
							Medium	35 (68.9%)	High	18 (35.3%)
							Medium-high	9 (17.6%)		
							High	5 (9.8%)		
CRI education	51	103 ± 11.2	97.0	106	0.942	0.015	Low	0 (0%)	Low	16 (31.4%)
							Medium-low	0 (0%)	Medium	17 (33.3%)
							Medium	41 (80.4%)	High	18 (35.3%)
							Medium-high	10 (19.6%)		
							High	0 (0%)		
CRI working activity	51	101 ± 19.5	89.7	105	0.932	0.006	Low	0 (0%)	Low	17 (33.3%)
							Medium-low	11 (21.6%)	Medium	16 (31.4%)
							Medium	27 (52.9%)	High	18 (35.3%)
							Medium-high	9 (17.6%)		
							High	4 (7.8%)		
CRI leisure time	51	108 ± 24.7	95.0	117	0.961	0.089	Low	2 (3.9%)	Low	15 (29.4%)
							Medium-low	7 (13.7%)	Medium	18 (35.3%)
							Medium	23 (45.1%)	High	18 (35.3%)
							Medium-high	9 (17.6%)		
							High	10 (19.6%)		

M, mean; N, number of participants; SD, standard deviation; p, probability value (significance level).

Kruskal-Wallis tests assessing equivalence across CR groups with respect to potential confounding variables showed no significant differences for any demographic or clinical variables for CRI Working Activity or CRI Leisure Time subdomains (all *p* > 0.05). In contrast, in CRI total a significant group difference emerged for diabetes (χ^2^ (2) = 7.02, *p* = 0.030), with DSCF comparisons indicating a difference between the low and high CR groups (*p* = 0.018). Similarly, CRI Education revealed a significant difference for physical activity (χ^2^ (2) = 6.93, *p* = 0.031), with DSCF comparisons indicating a difference between the medium and high CR groups (*p* = 0.033). Full results of Kruskal–Wallis tests and DSCF *post-hoc* comparisons are reported in the Supplementary material.

### 3.4 Differences in cognitive and psychological performance across cognitive reserve levels

Full results of all analyses are provided in the Supplementary material. The present section reports only domain-specific differences across CR levels that remained significant after correction for multiple comparisons at the domain level using the Benjamini-Hochberg procedure, together with the corresponding *post-hoc* comparisons. No significant differences were observed for global cognition, language, short-term memory, working memory, long-term memory domains, or subjective cognitive complaints, regardless of whether CR was operationalized using the CRI total score or its subdomains. Furthermore, Spearman correlations revealed no significant associations between any CR index and either CFI or MASCoD scores, converging with the group comparison results reported above.

#### 3.4.1 Attention and executive functions

**Figure 1:**
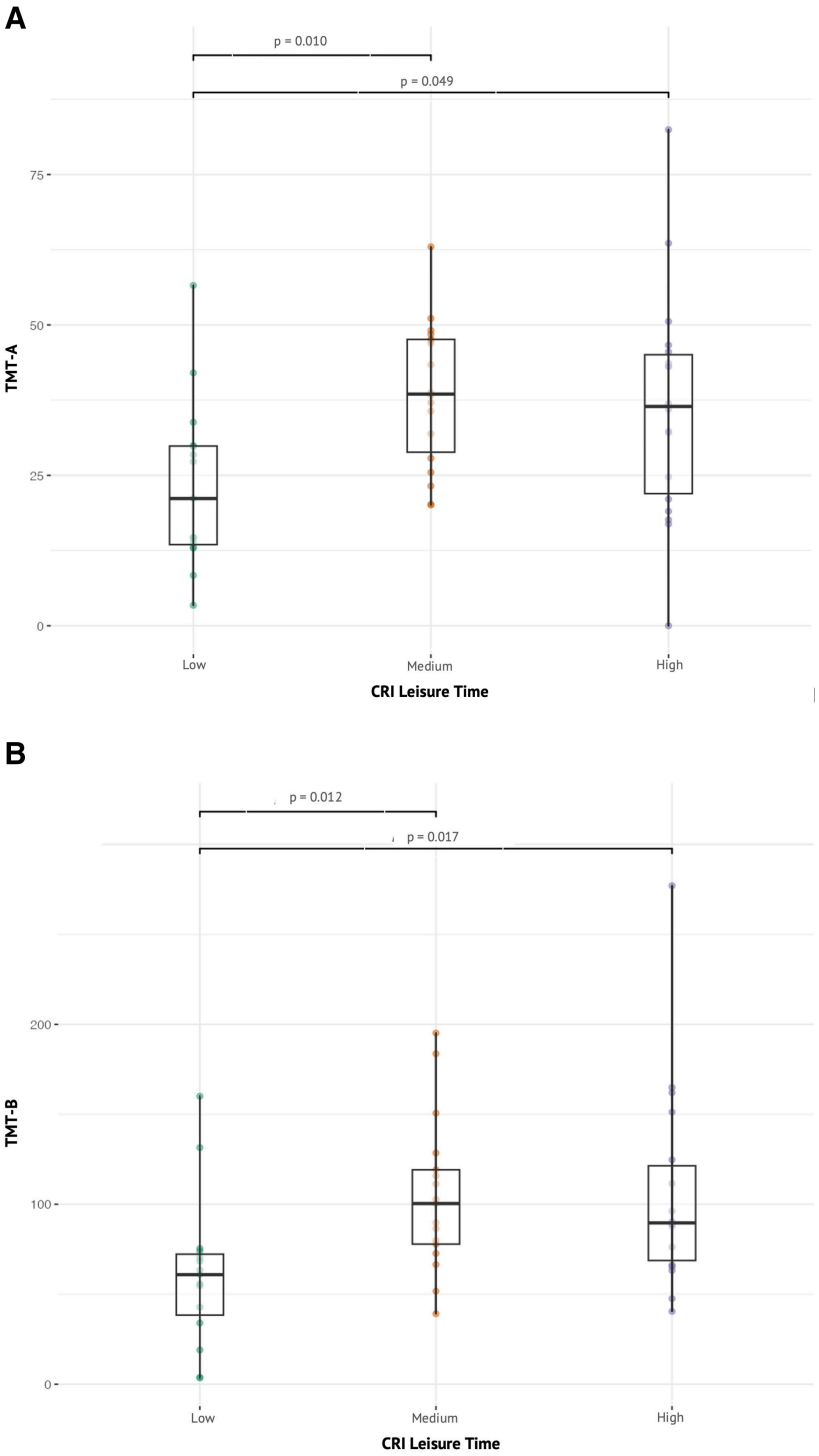
Boxplots of trail making test completion times across levels of CRI leisure time sub-domain. Boxes represent the interquartile range, the horizontal line indicates the median, and whiskers denote the data range. Significant pairwise differences are indicated. **(a)** TMT-A; **(b)** TMT-B.

When CR was operationalized using the CRI Leisure Time subdomain, significant group differences were observed for both TMT-A (χ^2^(2)=9.69, *p* = 0.008, p_FDR = 0.02, ε^2^=0.1938) and TMT-B (χ^2^(2)=10.36, p = 0.006, p_FDR = 0.02, ε^2^ = 0.2115), indicating large effect sizes. *Post-hoc* DSCF comparisons showed that individuals in the low leisure-related CR group exhibited significantly shorter completion times on both TMT-A and TMT-B compared to the medium (TMT-A: W = 4.14, *p* = 0.010; TMT-B: W = 4.03, *p* = 0.012) and high CR groups (TMT-A: W = 3.32, *p* = 0.049; TMT-B: W = 3.89, *p* = 0.017), whereas no significant differences were observed between the medium and high CR groups for either task (TMT-A: W = −1.16, *p* = 0.689; TMT-B: W = −0.47, *p* = 0.942). These group differences are illustrated in Figure 1a (TMT-A) and Figure 1b (TMT-B). Full statistical results are reported in the Supplementary material.

#### 3.4.2 Constructive praxis and visuospatial abilities

**Figure 2:**
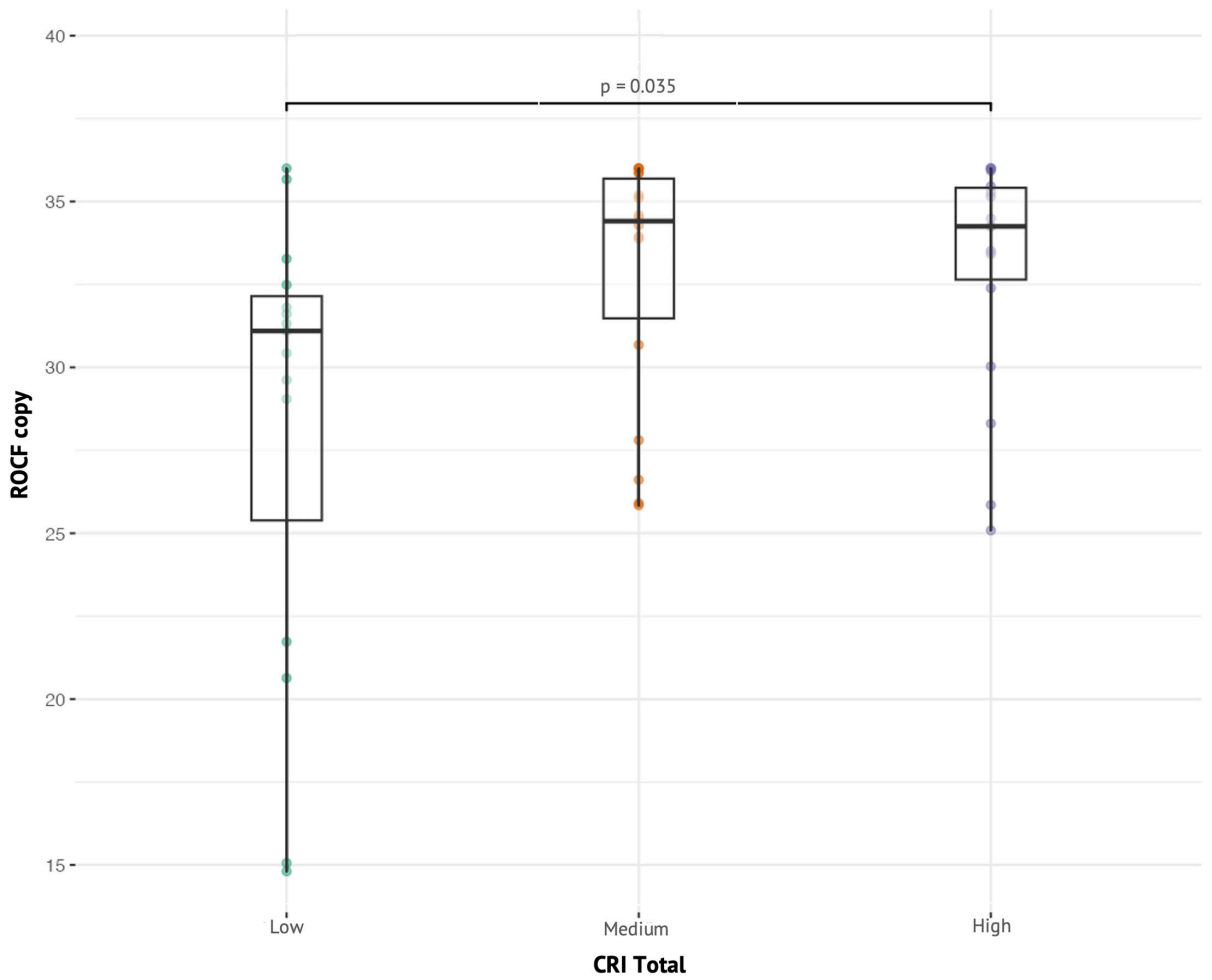
Boxplots of Rey–Osterrieth complex figure copy (ROCF copy) performances across levels of CRI Total. Boxes represent the interquartile range, the horizontal line indicates the median, and whiskers denote the data range. Significant pairwise differences are indicated.

When CRI total was considered, a significant group difference was observed for both ROCF copy (χ^2^(2)=7.48, *p* = 0.024, p_FDR = 0.048, ε^2^=0.1496), with a large effect size. *Post-hoc* DSCF comparisons showed poorer performance in the low CR group compared to the high CR groups (W = 3.51, *p* = 0.035), whereas differences between the low and medium CR groups did not reach statistical significance (W = 3.25, *p* = 0.056), and no difference was observed between the medium and high CR groups (W = −0.34, *p* = 0.969). This group difference is illustrated in Figure 2. Full statistical results are reported in the Supplementary material.

#### 3.4.3 Psychological functioning

**Figure 3:**
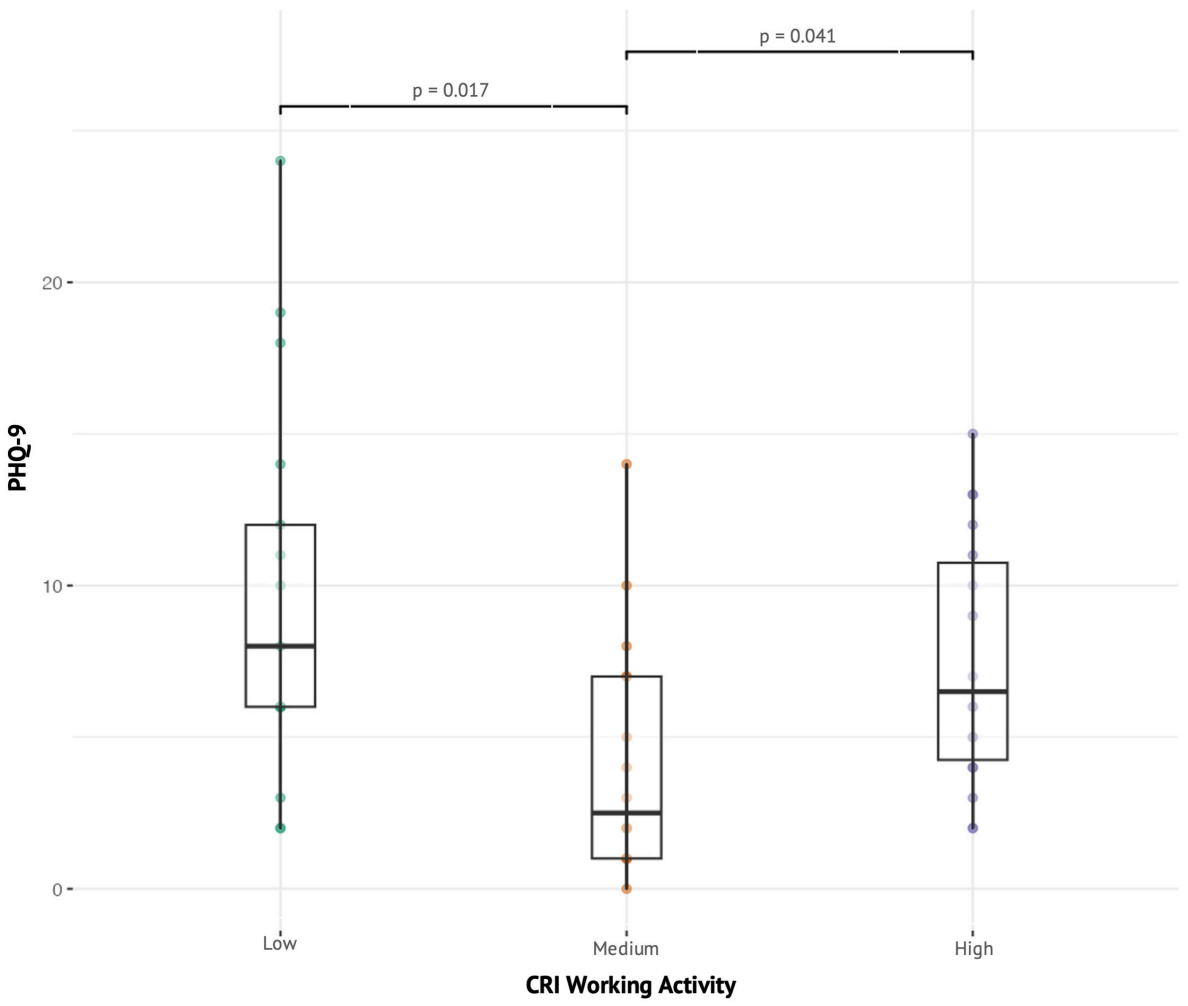
Boxplots of patient health questionnaire-9 (PHQ-9) performances across levels of CRI working activity. Boxes represent the interquartile range, the horizontal line indicates the median, and whiskers denote the data range. Significant pairwise differences are indicated.

Among the tests to assess psychological functioning, a significant group difference emerged only for depressive symptoms when CR was operationalized using the CRI Working Activity subdomain (χ^2^(2) = 9.19, *p* = 0.010, *p*<*uscore*>*FDR* = 0.04, ε^2^ = 0.1838), with a large effect size. *Post-hoc* DSCF comparisons showed that individuals in the medium CR group exhibited significantly lower PHQ-9 scores compared to both the low (W = −3.86, *p* = 0.017) and high CR groups (W = 3.43, *p* = 0.041), whereas no significant differences were observed between the low and high CR groups (W = −1.08, *p* = 0.726). This group difference is illustrated in Figure 3. Full statistical results are reported in the Supplementary material.

## 4 Discussion

### 4.1 Main findings summary

The present study examined how different components of CR, operationalized through the s-CRIq, relate to cognitive and psychological functioning in a clinical sample of individuals with SCD. In line with recent contributions, these findings support the view of SCD as a heterogeneous condition that must be understood by integrating sociodemographic, neuropsychological, psychological, and reserve-related factors to better characterize early vulnerability and resilience mechanisms in cognitive aging (Akbayrak et al., 2024; Jessen et al., 2014, 2020; Munro et al., 2023; Molinuevo et al., 2017; Rabin et al., 2017). Given the exploratory design, the discussion is organized around four main, interconnected aspects: (i) sociodemographic and clinical characteristics of the sample, including lifestyle profiles; (ii) cognitive and psychological features, with particular attention to the interplay between subjective complaints and affective symptoms; (iii) methodological implications of the assessment tools and reserve operationalization, especially in relation to ceiling effects and context-sensitive CR metrics; and (iv) emerging patterns linking different CR subdomains with specific cognitive and affective outcomes, with the aim of delineating hypotheses and priorities for future research. Moreover, given the cross-sectional design and the use of proxy measures of CR, the present study is explicitly framed at the level of associations rather than mechanisms: it includes CR proxies and cognitive outcomes but, lacking direct measures of brain change (e.g., neuroimaging or fluid biomarkers) and a longitudinal dimension, it is not possible to establish if and how CR can moderate the relationship between brain change and cognitive change (Stern et al., 2023).

#### 4.1.1 Sociodemographic and clinical characteristics

The present sample, despite being small and monocentric, comprised older adults with SCD recruited in a real-world clinical setting from an Italian CCDD. Participants were predominantly female, with a mean age of approximately 70 years and intermediate educational attainment. This profile appears broadly consistent with national demographic data for older Italian adults (Röhr et al., 2020). However, it only partially overlaps with international SCD cohorts (Röhr et al., 2020; Tank et al., 2024).

Particularly, the female predominance observed in this cohort can be considered consistent with epidemiological evidence from both national and international cohorts (Röhr et al., 2020; Tank et al., 2024). This pattern is especially relevant given that approximately two-thirds of AD diagnoses occur in women and that SCD is increasingly conceptualized as a clinical at-risk state along the AD continuum (Jessen et al., 2014; Kang et al., 2024; Wang et al., 2020). However, the mechanisms underlying these gender differences have yet to be clarified, and the hypotheses proposed to date require more in-depth investigation.

By contrast, the educational composition of the sample reflects that of older Italian cohorts, in which lower and intermediate education levels remain prevalent (Röhr et al., 2020). This pattern differs from that reported in several international SCD studies, where educational levels are generally higher (Röhr et al., 2020; Tank et al., 2024). This characteristic is particularly relevant in the context of CR, as education represents one of its most robust proxies and is consistently associated with better late-life cognitive functioning and reduced dementia risk (Arora et al., 2024; Ma et al., 2024; Stern, 2012; Stern et al., 2020). In the present cohort, the restricted educational range may have reduced variability in CR and promoted clustering within intermediate CR levels.

Finally, from a lifestyle and clinical perspective, the cohort showed low rates of hyperuricemia, psychoactive substance misuse, and alcohol consumption (Livingston et al., 2020, 2024). In the present study, some differences in diabetes and physical activity—both recognized correlates of SCD and of cognitive aging more broadly (Hao et al., 2026; Zülke et al., 2026)—emerged across CR strata. These findings appear broadly consistent with previous European SCD cohorts (Tank et al., 2024), but these variables were not included as covariates because the exploratory design and modest sample size limited the robustness of multivariable models. Representative trends emerged also for the proportion of current smokers, regular alcohol consumers and positive family history for neurocognitive disorders (Tank et al., 2024). More distinctive is the high burden of vascular risk factors which, although typical of older clinical populations, is relevant for SCD and may be even more pronounced here than in some larger international cohorts (Livingston et al., 2020, 2024; Tank et al., 2024). This is important because recent evidence converges on the idea that SCD reflects not only early neurodegenerative changes but also the interplay of vascular, psychological and lifestyle factors (Jessen et al., 2020; Li et al., 2023; Liew, 2020a; Ma et al., 2024; Ribaldi et al., 2022). In line with this, Guan et al. (2025) showed that having three or more vascular risk factors significantly increases the odds of reporting SCD, underscoring the central role of cardiovascular health in shaping both subjective complaints and objective cognition.

#### 4.1.2 Cognitive and psychological profile

A central and clinically relevant observation of the present study—as well as of SCD research in general—is the discrepancy between objectively preserved neuropsychological performance and noteworthy psychological vulnerability (Jessen et al., 2014; Viviano and Damoiseaux, 2020). All participants performed significantly above established clinical cut-offs across neuropsychological measures, indicating intact cognitive functioning fully consistent with SCD diagnostic criteria (Facchin et al., 2022; Hill et al., 2016, 2021; Zlatar et al., 2018).

Notwithstanding this preserved cognitive profile, a proportion of participants reported subjective difficulties—as captured by both the CFI and the MASCoD. At the group level, depressive symptomatology exceeded the threshold for at least mild depressive symptoms, whereas anxiety scores did not significantly differ from normative values. This pattern suggests that depressive symptoms—rather than anxious ones—may contribute to shaping the subjective experience of decline among cognitively preserved older adults with SCD. Indeed, existing evidence indicates that depression often shows a more consistent linear relationship with subjective complaints and cognitive outcomes, whereas anxiety effects tend to be weaker, curvilinear, or more context-dependent (Desai et al., 2021). However, the age of the sample adds a layer of interpretive complexity. Some of the symptoms captured by the self-report measures of affective functioning used in this study may overlap with age-related biological changes or comorbid medical conditions (Devita et al., 2022). Therefore, they should not be interpreted as direct evidence of a mood or anxiety disorder. At the same time, this does not make these symptoms clinically irrelevant. Rather, it suggests that elevated scores should be interpreted as indicators of a broader subjective symptom burden, emerging at the intersection between physical health, aging-related changes, and psychological functioning (Husain-Krautter and Ellison, 2021). From this perspective, the exclusion of individual with current or past DSM-5-TR psychiatric diagnosis does not preclude the presence of subthreshold psychological distress. In this sample, therefore, depressive and anxiety symptoms may reflect a clinically meaningful dimension of vulnerability (Devita et al., 2022) that, even in the absence of a formal psychiatric diagnosis, can shape both the subjective experience of cognitive difficulties and the overall psychological profile of individuals with SCD. This is particularly relevant when interpreting the mechanisms underlying subjective complaints, and it underscores the need for systematic psychological assessment within the clinical evaluation of SCD. Consistent with these considerations, a growing body of evidence indicates that subjective cognitive complaints may be more strongly associated with mood disturbances than with concurrent objective cognitive performance (Hill et al., 2021; Sabatini et al., 2022; Zlatar et al., 2018).

Consistent with this view, initial finding suggest that depressive symptoms may not only intensify complaints but also modulate how moderated the association between executive functioning and subjective complaints, suggesting a possible role of executive processes in shaping cognitive complaints (Maffoni et al., 2025b). Converging findings have been reported by Hill et al. (2016, 2021)—who found that depressive symptoms can mediate the relationship between SCD and memory functioning over time—and by Liew (2019) –who proposed that mood disorders may act both as antecedents and consequences of subjective cognitive concerns.

From a clinical perspective, this evidence is highly relevant. Indeed, as outlined in the introduction, seminal work by Jessen et al. (2014, 2020) and subsequent longitudinal studies have delineated heterogeneous SCD trajectories, each associated with different risks of progression to MCI or dementia (Mitchell et al., 2014; Slot et al., 2019; Ribaldi et al., 2022). Persistent SCD appears to confer the highest risk of clinical conversion, whereas transient or fluctuating complaints are more frequently linked to potentially reversible psychological or contextual factors, including depressive symptoms, stress, or bereavement (Amariglio, 2021; Hill et al., 2016). In this light, the pattern observed in our cohort may be interpreted as being compatible with a subgroup in which affective factors contribute substantially to the manifestation and persistence of complaints, even in the absence of measurable cognitive impairment.

Taken together, both the present findings and the broader literature underscore that subjective cognitive complaints, in isolation, are insufficient to infer underlying neuropathology. Instead, they must be interpreted within a multidimensional framework that integrates objective cognitive performance, mood and anxiety symptoms, vascular and clinical risk factors, and—where feasible—biomarkers and neuroimaging measures (Molinuevo et al., 2017; Frisoni et al., 2024).

#### 4.1.3 Assessment strategies and their methodological and clinical implications

The ceiling effects observed across cognitive measures have important methodological implications for research on CR in the early stages of cognitive aging. Most notably, such effects constrain variability in outcome measures, thereby attenuating statistical power and limiting the sensitivity of analyses to detect meaningful associations or group differences (Jester et al., 2021). When most participants perform near the top of the score range, group comparisons are likely to underestimate true differences because of insufficient variability. This phenomenon could partly explain why, in the present sample, significant differences emerged mainly on more demanding tasks (e.g., ROCF Copy, TMT-A, TMT-B) and within dynamic CR subdomains, rather than across all assessed domains. Consequently, future studies on SCD populations should consider using more sensitive or higher-difficulty cognitive measures, better suited to capturing subtle reserve-related effects (De Simone et al., 2023; Marra et al., 2021).

A similar issue of restricted variability emerged in the distribution of CR scores. Actually, one of the main methodological contributions of this study lies in documenting the mismatch between the normative categorization scheme for the s-CRIq (Mondini et al., 2023) and the actual distribution of scores in the present sample. In the current data, standard s-CRIq categories produced a highly skewed distribution, with most participants clustering in the medium range. This pattern is likely related to the limited variability in educational attainment and the relatively homogeneous occupational background of this Italian cohort, which differs from the broader and more socioeconomically diverse normative sample used to derive the s-CRIq cut-offs (Mondini et al., 2023; Nucci et al., 2012). In contrast, leisure-time activities showed a less skewed distribution, possibly because they are less constrained by past educational opportunities and therefore can vary more flexibly across the life course. This methodological issue is also evident in cross-cultural CR research. Suemoto et al. (2022), analyzing education and occupation as reserve proxies in a large Brazilian autopsy cohort, found that normative categorizations derived in high-income countries did not adequately fit their sample, where 77% of participants had fewer than 5 years of education and 56% held unskilled occupations. Hence, studies should carefully consider whether existing CR measures and cut-offs are appropriate for the sociocultural and educational context under investigation, particularly in settings that resemble the present cohort in terms of limited educational variability and relatively homogeneous occupational profiles. In such contexts, adapting CR indices may be necessary to obtain valid and informative comparisons.

#### 4.1.4 Emerging pattern in cognitive reserve and cognitive-affective outcomes

Among the multiple cognitive domains assessed, CRI Total showed a specific association with visuospatial-constructional performance. Individuals with high CRI Total obtained significantly higher ROCF Copy scores than those with low CRI Total, while differences between low and medium CRI Total were only marginal. First of all, it should be pointed out that a difference emerged despite the overall ceiling effect in ROCF Copy performance in this cognitively normal sample. This could indicate that even among cognitively preserved individuals without objective impairment, CR continues to account for performance variation in demanding tasks (Barulli and Stern, 2013). Secondly, these results suggest a possible threshold effect, whereby moderate-to-high levels of reserve are associated with better visuospatial-constructional performance, whereas low reserve is associated with relatively poorer performance in this domain. This finding may be consistent with emerging evidence from neuroimaging and experimental studies suggesting that CR can be most strongly linked to higher-order cognitive operations requiring planning, strategic organization, and flexible network recruitment rather than basic sensorimotor processes (Cabeza et al., 2018; Mauti et al., 2025). Indeed, the ROCF Copy task is a complex cognitive activity rather than a purely perceptual or motor exercise (Frigeni et al., 2024). Optimal performance requires the coordinated integration of visuospatial analysis, executive planning and organizational strategies, working memory to maintain spatial relationships, graphomotor execution, and continuous monitoring with error correction (Frigeni et al., 2024; Lingo VanGilder et al., 2020). These processes engage widely distributed cortical networks (Frigeni et al., 2024; Lingo VanGilder et al., 2020). Experimental work on visuospatial cognition in healthy older adults similarly indicates that higher reserve is most evident on demanding tasks that require strategic organization and flexible network recruitment. For example, studies using graded-difficulty mental rotation paradigms report that higher CR predicts better accuracy on complex spatial configurations, whereas effects on basic speed measures are minimal or absent (Mauti et al., 2025). Although this framework provides a plausible interpretive context for the observed associations, direct testing was beyond the scope of this cross-sectional study. The analysis was limited to examining relationships between cognitive reserve proxies and cognitive performance and therefore does not offer direct evidence of compensatory or resilience mechanisms.

Within this framework, the pattern observed for leisure-time CR and processing speed becomes more interpretable, although it could remain apparently unexpected. In the present sample, individuals with lower leisure-time CR completed both TMT-A and TMT-B faster than those with medium or high leisure-time CR—contrary to the conventional expectation that higher reserve is uniformly associated with better performance (Stern et al., 2020). This pattern suggests that processing speed may not be a domain in which CR exerts strong protective effects. Rather, TMT completion times may be more closely related to neurobiological substrates – such as white-matter integrity and neural transmission efficiency, as suggested by external neuroimaging evidence (MacPherson et al., 2017; Miskin et al., 2016)—than to reserve-related factors captured by CR proxy measures. Indeed, this interpretation is supported by neuroimaging evidence linking slower TMT-B performance to cortical thinning and reduced white-matter integrity in fronto-temporal networks, with these associations largely attenuated after accounting for processing speed itself (MacPherson et al., 2017; Miskin et al., 2016). Converging evidence comes from Mauti et al. (2025), who showed that higher CR was associated with greater accuracy on complex visuospatial tasks, but not with faster response times. Taking together these studies and the results of the present investigation, results suggest that CR proxy measures may be more strongly associated with higher-order, strategy-dependent performance than with processioning speed, which appears more closely linked to biological aging trajectories. However, causal interpretations are again prevented by the cross-sectional design and by the nature of the data collected.

Finally, the data suggest a significant non-monotonic association between occupational CRI Working Activity and depressive symptoms—again contrary to the usual protective effect proposed by CR theory (Stern, 2012; Stern et al., 2020). Indeed, this association followed an inverted-U pattern: individuals with moderate occupational complexity reported fewer depressive symptoms than those with either low or high complexity, while no significant differences emerged between the latter two groups. This pattern is coherent with the Yerkes-Dodson law and related models in occupational health psychology, which state that while under stimulation and excessive demands are associated to worse mental health outcomes, moderate levels of cognitive and psychological demand are associated with better wellbeing (Yerkes and Dodson, 1908). Low occupational complexity may indicate lack of opportunities and cognitive challenges, which could make people more susceptible to disengagement and depressive symptoms, especially after retirement (Stansfeld and Candy, 2006; Butterworth et al., 2009). At the other extreme, prolonged exposure to highly demanding occupational tasks may be linked to cognitive overload, chronic stress, and burnout, with psychological consequences that may persist after active work (Festini, 2022; Salvagioni et al., 2017; Stansfeld and Candy, 2006). Hence, the group with moderate occupational complexity may provide the optimal balance between appropriate demands and consistent cognitive engagement. Furthermore, occupational complexity may reflect cumulative lifetime job exposure rather than current employment engagement because the majority of participants in the current sample were retired, highlighting the possible long-term mental health consequences of occupational histories. These findings should be interpreted with caution, but they suggest that the relationship between occupational cognitive demands and late-life depressive symptoms may not be adequately captured by simple linear models of CR.

### 4.2 Strengths, limitations and future research

This exploratory study has several strengths. First, rather than using a single proxy, CR was operationalized and regarded as a multidimensional construct, distinguishing education attainment, occupational complexity, and leisure-time activities. By evaluating these elements independently using the s-CRIq, it was possible to identify differential associations with cognitive and psychological outcomes, providing a more refined characterization of reserve mechanisms in SCD. Second, the inclusion of a thorough neuropsychological and psychological assessment made it possible to identify domain-specific reserve effects that may otherwise have remained undetected.

Nevertheless, some limits should be considered. First, the study's exploratory and cross-sectional methodology prevented the possibility to proceed with causal inference and did not allow definitive conclusions regarding the direction or size of the observed associations. Although certain relationships showed substantial effect sizes, statistical power and generalizability were hampered by the short sample size. Likewise, missing data on subjective complaint measures reduced statistical power for analyses involving these instruments as outcomes. Besides, outcome variability was limited by ceiling effects in cognitive performance, which may also have decreased sensitivity to identify reserve-related effects. The limited demographic heterogeneity of the sample, including a predominance of female participants and a restricted range of educational attainment, further constrained the applicability of the results to broader populations. Furthermore, although SCD status was established clinically in accordance with SCD-I Criteria, mean CFI scores fell below the normative cut-off at the group level. This is consistent with what is typically observed in real-world help-seeking clinical cohorts. Nevertheless, the present protocol did not assess for SCD-plus features, and the sample was consequently not stratified. Therefore, these findings may not fully generalize to individuals with more pronounced subjective complaints meeting SCD-plus criteria, who are considered at higher risk for underlying neurodegenerative disease. Another limitation concerns the assessment of psychological functioning: depressive and anxiety symptoms were measured with the PHQ-9 and the GAD-7, used as screening measures based on severity cut-offs. Thus, they are useful to quantify symptom severity but cannot, on their own, establish formal mood or anxiety disorders. This consideration is especially relevant in older adults, as some affective symptoms may overlay with age-related biological changes or comorbid medical conditions, rather than directly reflecting depression or anxiety. Moreover, the absence of neuroimaging data prevented the examination of the neural mechanisms underlying compensatory reserve processes. Finally, while depressive symptoms were examined as a primary outcome in the present study, their potential role as a moderator of the associations between CR and objective cognitive performance could not be formally tested given the sample size and the non-parametric analytical framework adopted.

Future studies should aim to extend these findings through longitudinal and multimodal approaches, with larger cohorts. First, larger samples would, in the first place, allow vascular and lifestyle risk factors—such as diabetes and physical activity, which here differed across CR strata—to be included as covariates within multivariable models, thereby disentangling their contribution from that of CR. Second, larger studies could enable also the possibility to explicitly examine whether CR-cognition associations remain after accounting for depressive symptomatology. To find out if baseline CR—both global and domain-specific—predicts the development of MCI or dementia, prospective studies tracking SCD people over time are crucial. In this regard, future work should also explicitly identify individuals meeting SCD-plus criteria and examine whether the domain-specific reserve patterns observed here differ in this higher-risk subgroup, given their greater likelihood of underlying preclinical AD. In order to evaluate the reserve compensation theory and determine if high reserve confers resilience despite larger neuropathological burden, it is likewise necessary to integrate biomarker data. Besides, in an effort to clarify neuronal mechanisms, complementary neuroimaging studies integrating structural, functional, and metabolic modalities will be necessary. Future work should emphasize sex-stratified analyses to address potential gender-specific mechanisms in how reserve modulates cognitive and affective outcomes. Finally, future research should move beyond association to explicitly formulate and test causal hypotheses regarding the relationships between specific CR subdomains and cognitive and affective outcomes. This further underscores the need for longitudinal and interventional designs capable of determining the directionality of these associations and disentangling reserve-building effects from selection mechanisms.

## 5 Conclusions

Given the exploratory nature of this study, the findings should be interpreted cautiously. Nonetheless, some informative patterns emerge. Despite a comprehensive neuropsychological assessment, CR showed few significant differences across most cognitive domains. This selective pattern may reflect specific features of how CR operates at the stage of cognitive aging represented by SCD, rather than necessarily indicating weak or absent reserve effects, although this interpretation remains tentative given the study's design. The findings suggest that reserve is not a unitary construct with uniform effects across cognition. Instead, different reserve dimensions may have domain-specific effects. Global reserve may be most strongly associated with visuospatial and strategic processing, leisure-related reserve may preferentially support attentional and executive functioning, and occupational history may be especially important for mood and psychological wellbeing. These domain-specific patterns are preliminary and require confirmation in larger, longitudinal studies. Overall, for precise risk assessment and clinical interpretation in SCD, it is important to comprehend both the general level of CR and the distinct contributions of its subdomains.

## Data Availability

The raw data supporting the conclusions of this article will be made available by the authors, without undue reservation.
