# Supplementary material for: Why some minds bend, not break: an exploratory study of cognitive and psychological performance across levels of cognitive reserve in subjective cognitive decline
Source: Front Psychol. 2026 Jun 26;17:1808150. doi: 10.3389/fpsyg.2026.1808150 (PMC13349775; doi:10.3389/fpsyg.2026.1808150)
Supplement: Supplementary file 1 [file Table_1.DOCX]

| **Descriptives sociodemographic variable** | | | | | | | | | | | |
| --- | --- | --- | --- | --- | --- | --- | --- | --- | --- | --- | --- |
|  | | | | | | **Skewness** | | **Kurtosis** | | **Shapiro-Wilk** | |
|  | **N** | **Missing** | **Mean** | **Median** | **SD** | **Skewness** | **SE** | **Kurtosis** | **SE** | **W** | **p** |
| **Age** | 51 | 0 | 69.73 | 72 | 9.3168 | -0.5828 | 0.333 | -0.310 | 0.656 | 0.954 | 0.048 |
| **Years of schooling** | 51 | 0 | 10.31 | 10 | 3.8910 | 0.3237 | 0.333 | -0.604 | 0.656 | 0.927 | 0.004 |
| **Weight (kg)** | 43 | 8 | 70.72 | 72 | 13.6405 | 0.0820 | 0.361 | -0.928 | 0.709 | 0.963 | 0.179 |
| **Height (m)** | 43 | 8 | 1.64 | 1.62 | 0.0841 | 0.1304 | 0.361 | -0.433 | 0.709 | 0.944 | 0.036 |
| **BMI** | 43 | 8 | 26.32 | 25.00 | 5.1535 | 1.3898 | 0.361 | 3.339 | 0.709 | 0.905 | 0.002 |

| 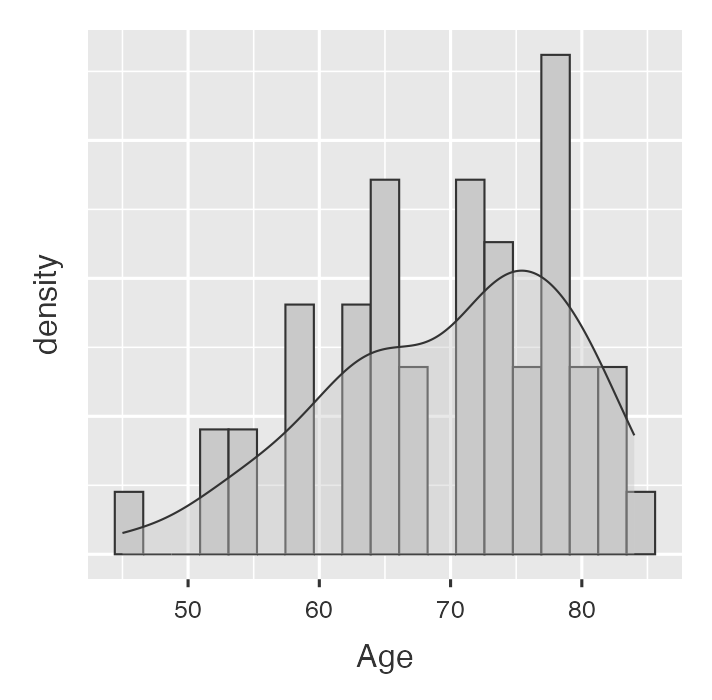 | 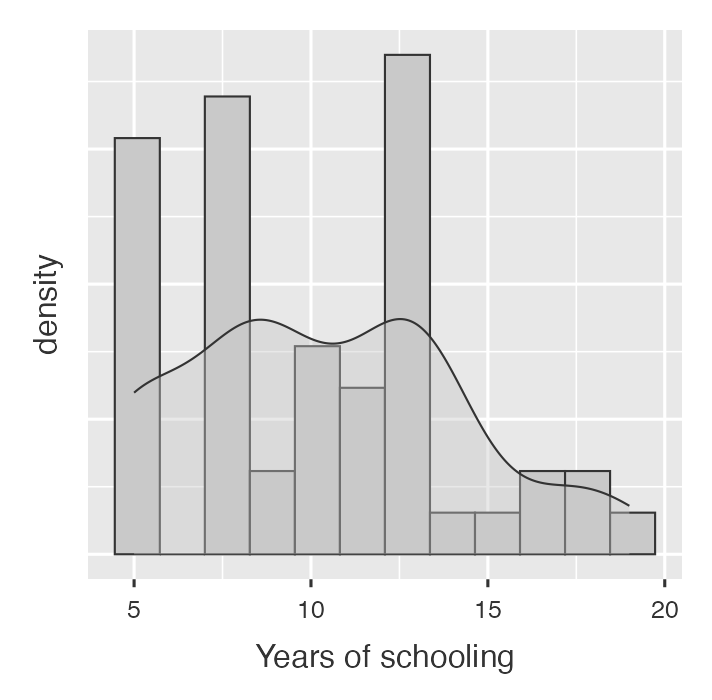 | 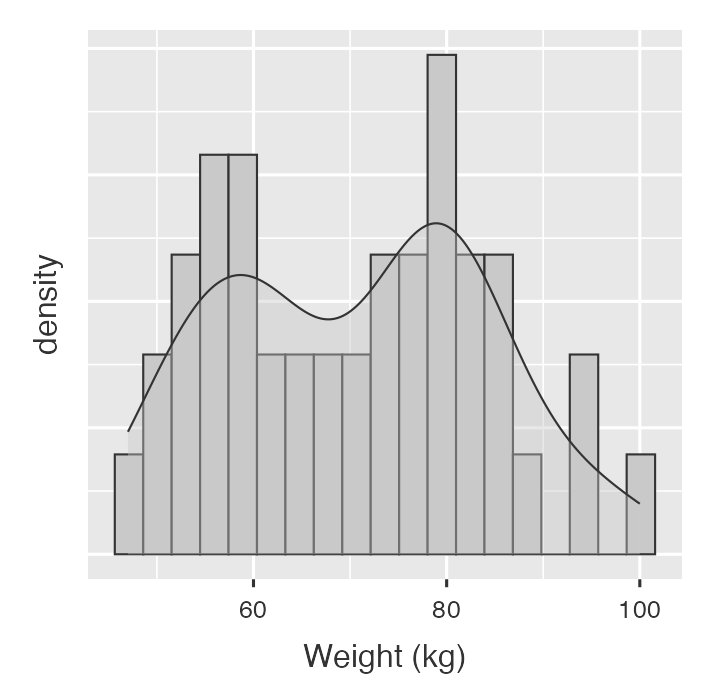 |
| --- | --- | --- |
| 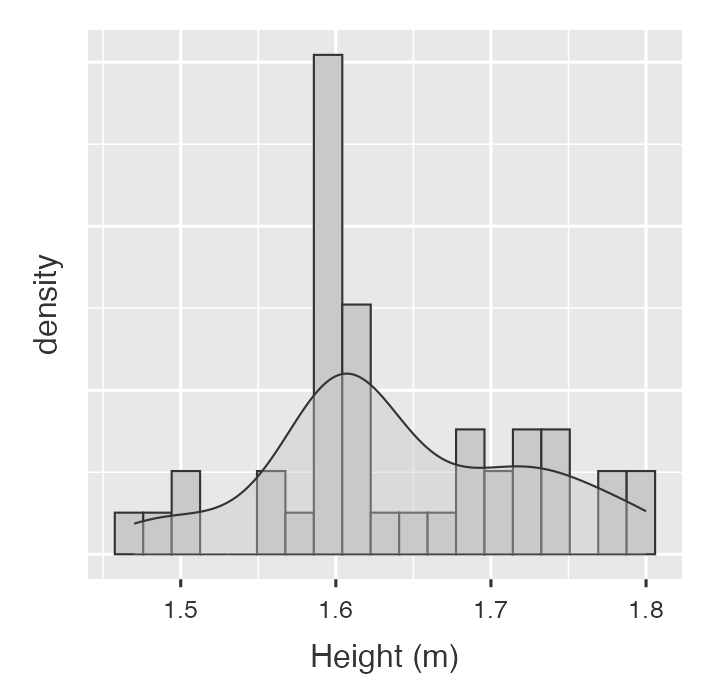 | 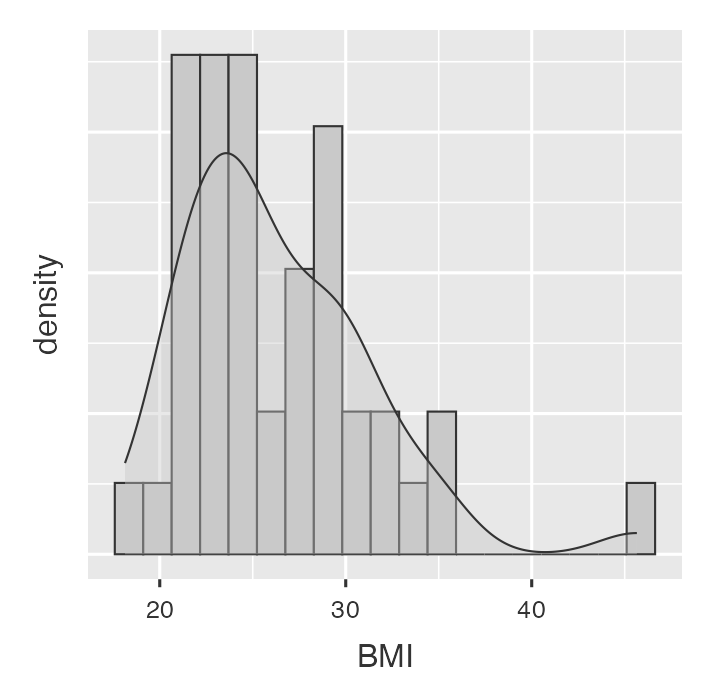 |  |

| **Descriptives cognitive variables** | | | | | | | | | | | | | |  |  |
| --- | --- | --- | --- | --- | --- | --- | --- | --- | --- | --- | --- | --- | --- | --- | --- |
|  | | | | | | | **Skewness** | | **Kurtosis** | | **Shapiro-Wilk** | | |  |  |
|  | | **N** | **Missing** | **Mean** | **Median** | **SD** | **Skewness** | **SE** | **Kurtosis** | **SE** | **W** | **p** | | |  |
| **MMSE** | 51 | | 0 | 28.977 | 29.43 | 1.262 | -0.9565 | 0.333 | -0.542 | 0.656 | 0.806 | | <.001 | | |
| **ACE_III** | | 44 | 7 | 87.086 | 87.68 | 7.557 | -0.2342 | 0.357 | -0.756 | 0.702 | 0.974 | | 0.416 | | |
| **FAB** | | 51 | 0 | 15.136 | 15.12 | 2.233 | -0.3216 | 0.333 | -0.771 | 0.656 | 0.961 | | 0.089 | | |
| **ROCF_Copy** | | 51 | 0 | 31.605 | 33.52 | 5.164 | -1.7097 | 0.333 | 2.838 | 0.656 | 0.796 | | <.001 | | |
| **ROCF_Delayed** | | 51 | 0 | 16.063 | 16.71 | 6.840 | -0.0496 | 0.333 | -0.433 | 0.656 | 0.989 | | 0.929 | | |
| **DSF** | | 51 | 0 | 5.610 | 5.45 | 0.918 | 0.4953 | 0.333 | -0.307 | 0.656 | 0.971 | | 0.240 | | |
| **DSB** | | 51 | 0 | 4.245 | 4.24 | 1.047 | -0.5741 | 0.333 | 6.867 | 0.656 | 0.873 | | <.001 | | |
| **Phonemic_Fluency** | | 51 | 0 | 36.274 | 35.52 | 9.050 | 0.2083 | 0.333 | 0.139 | 0.656 | 0.986 | | 0.815 | | |
| **Semantic_Fluency** | | 50 | 1 | 46.979 | 46.85 | 10.169 | 1.1135 | 0.337 | 2.455 | 0.662 | 0.929 | | 0.005 | | |
| **CSF** | | 51 | 0 | 5.432 | 5.50 | 1.332 | -1.0800 | 0.333 | 4.517 | 0.656 | 0.925 | | 0.003 | | |
| **CSB** | | 51 | 0 | 4.718 | 4.91 | 1.479 | -1.1147 | 0.333 | 2.372 | 0.656 | 0.914 | | 0.001 | | |
| **TMT_A** | | 51 | 0 | 33.346 | 32.32 | 16.478 | 0.3764 | 0.333 | 0.387 | 0.656 | 0.982 | | 0.635 | | |
| **TMT_B** | | 50 | 1 | 91.643 | 83.18 | 50.931 | 1.1580 | 0.337 | 2.529 | 0.662 | 0.930 | | 0.006 | | |
| **CDT** | | 51 | 0 | 56.290 | 58.33 | 7.628 | -2.7049 | 0.333 | 7.193 | 0.656 | 0.617 | | <.001 | | |
| **RAVLT_Immediate** | | 51 | 0 | 45.208 | 43.38 | 9.458 | 0.3897 | 0.333 | -0.305 | 0.656 | 0.973 | | 0.290 | | |
| **RAVLT_Delayed** | | 51 | 0 | 9.917 | 10.14 | 3.819 | -0.6482 | 0.333 | 0.169 | 0.656 | 0.961 | | 0.096 | | |
| **STROOP_Time** | | 49 | 2 | 16.870 | 15.28 | 7.903 | 0.6785 | 0.340 | -0.193 | 0.668 | 0.942 | | 0.017 | | |
| **STROOP_Errors** | | 49 | 2 | 0.811 | 0.00 | 2.312 | 3.4969 | 0.340 | 13.052 | 0.668 | 0.410 | | <.001 | | |

| 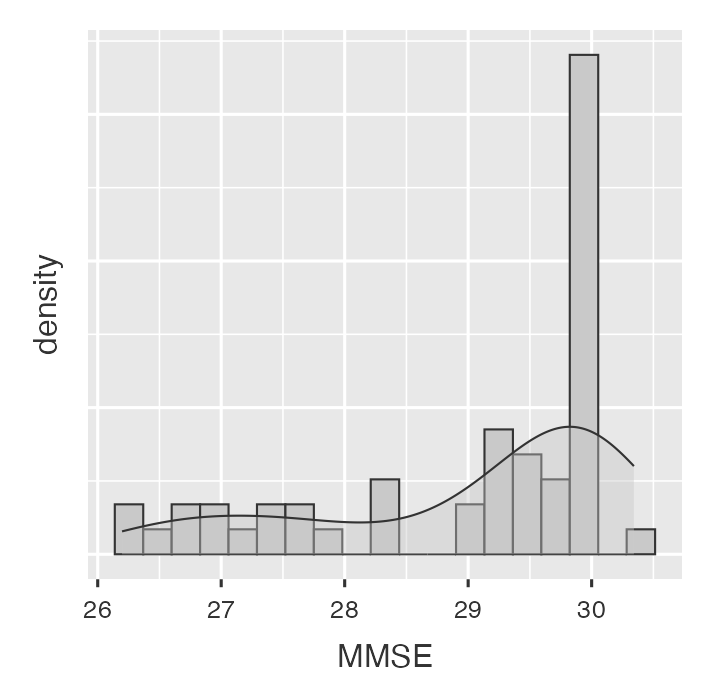 | 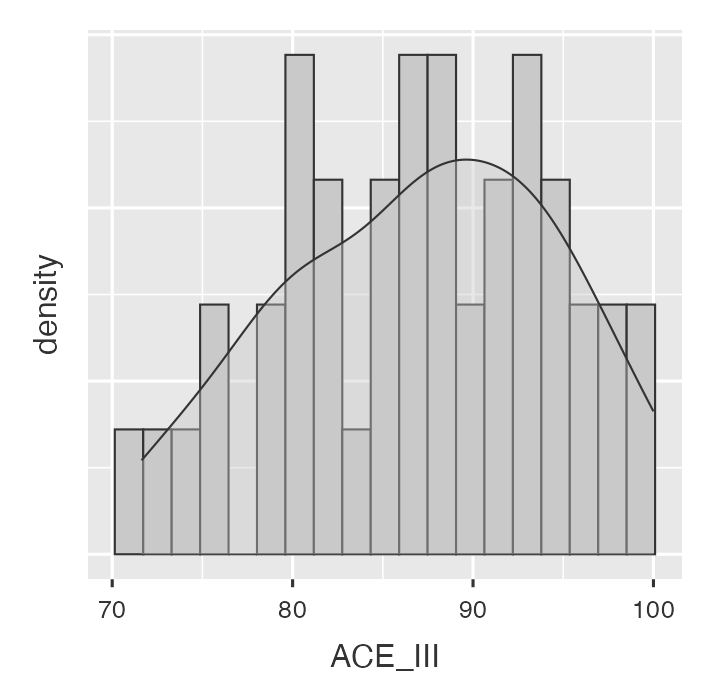 | 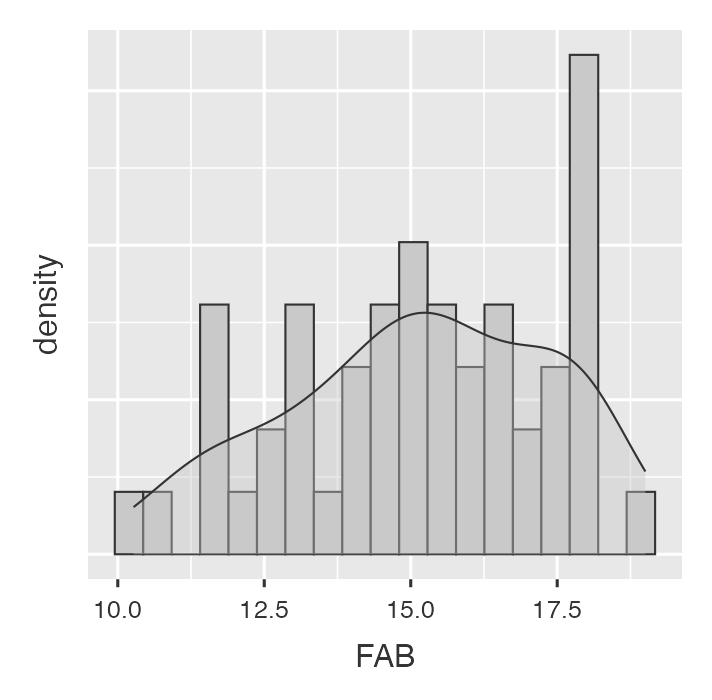 |
| --- | --- | --- |
| 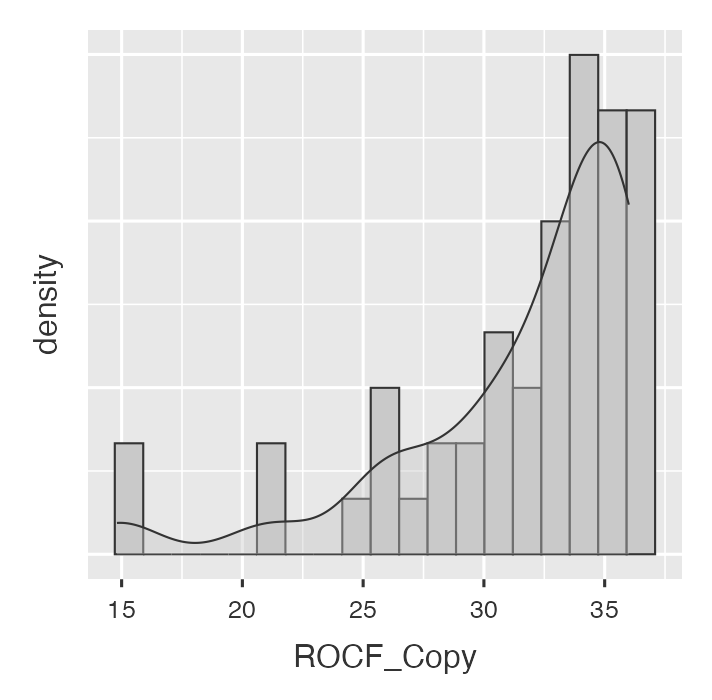 | 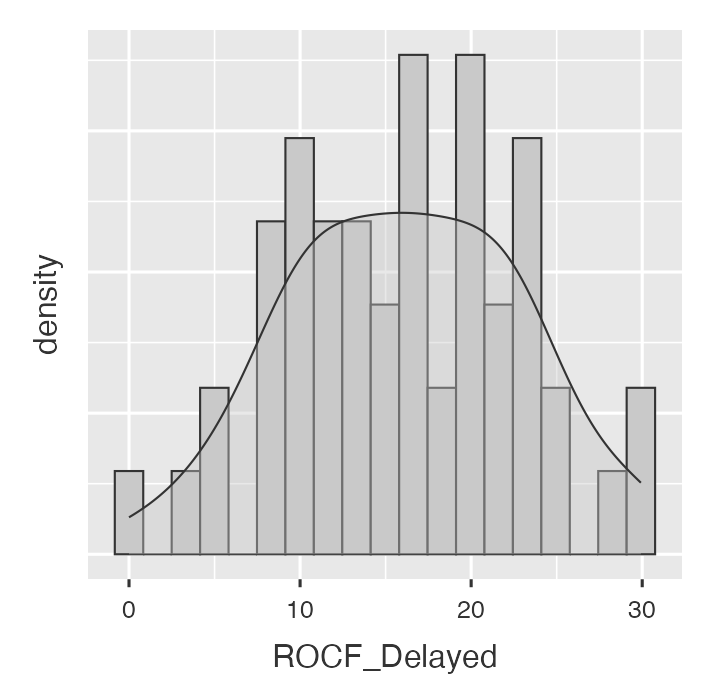 | 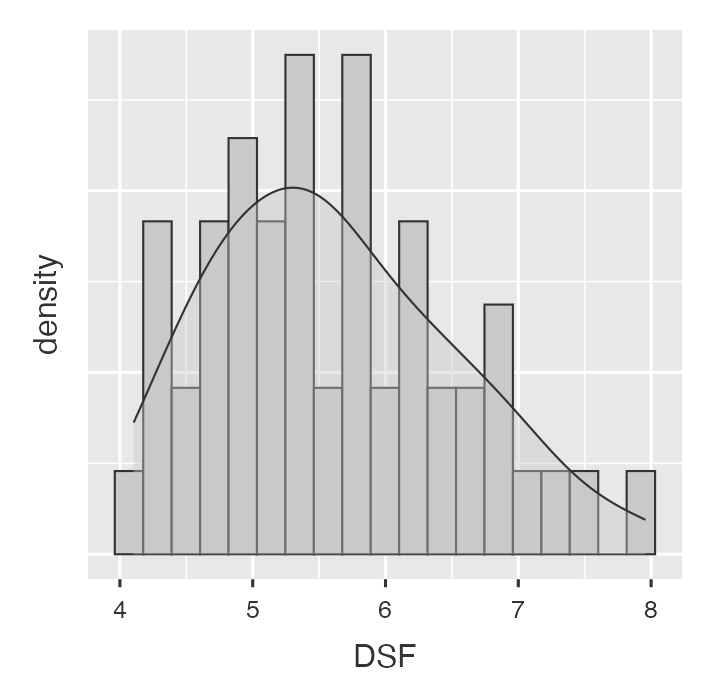 |
| 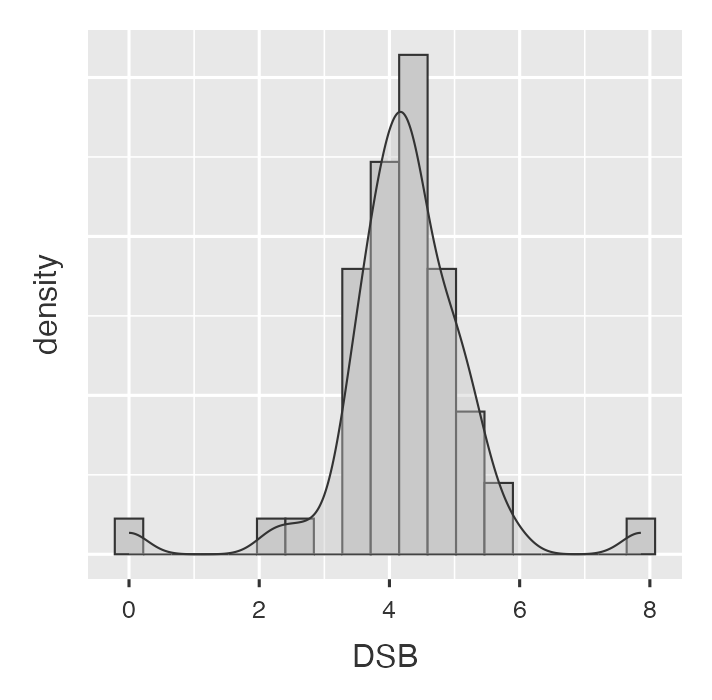 | 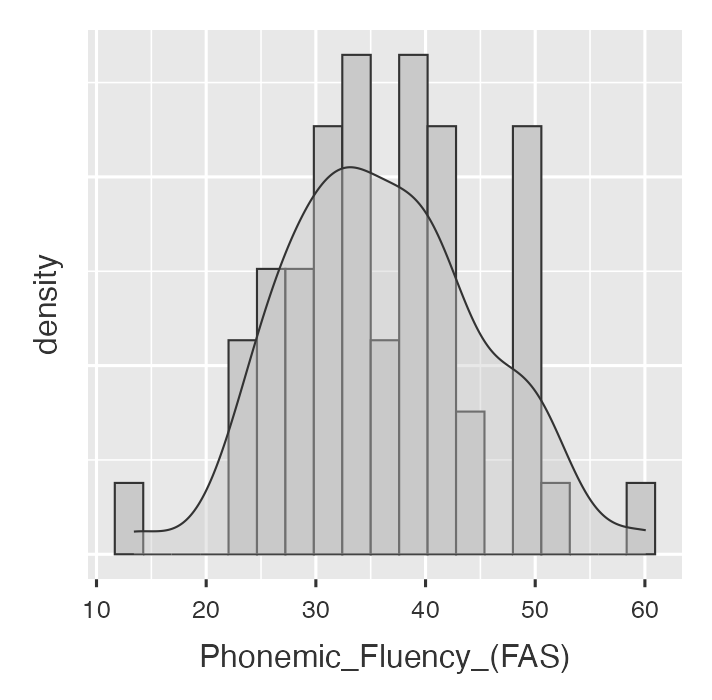 | 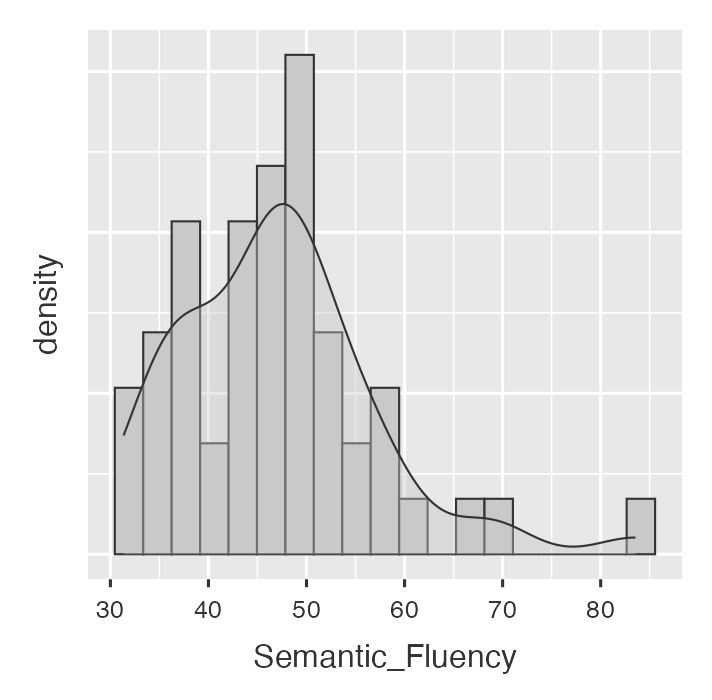 |
| 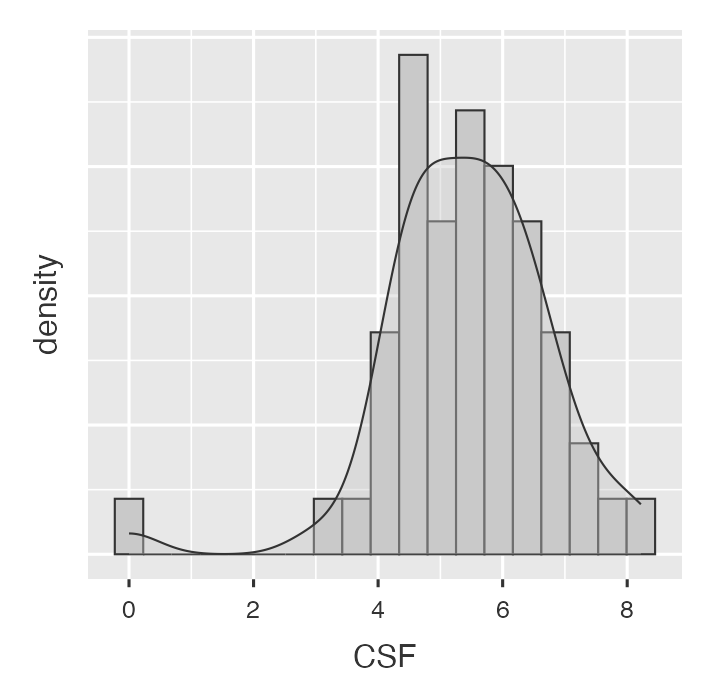 | 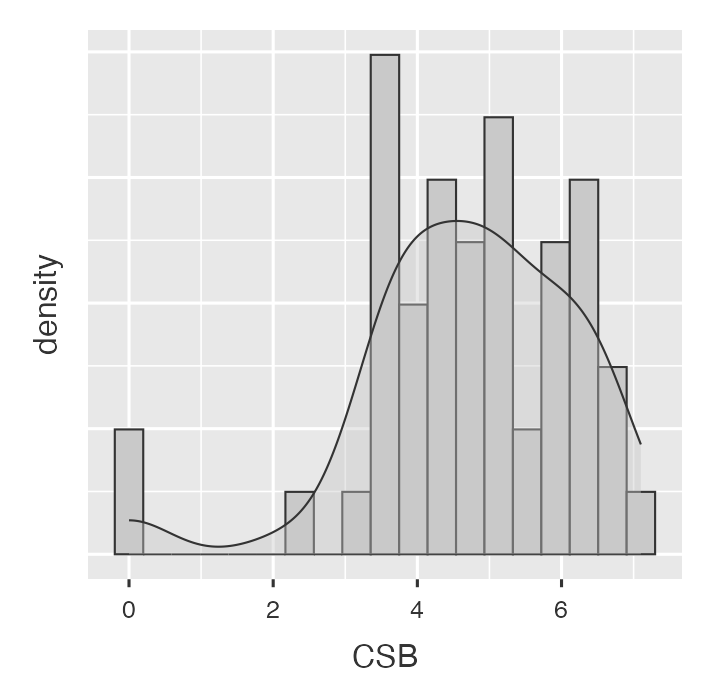 | 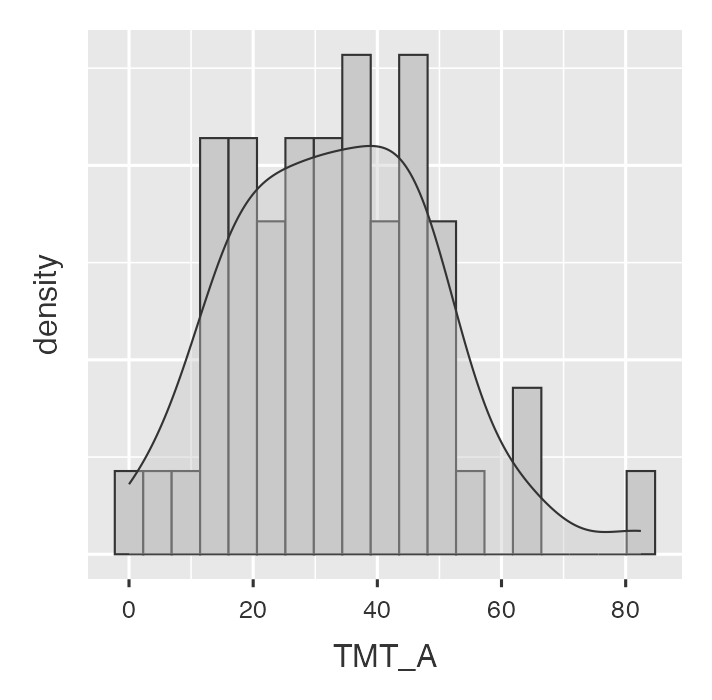 |
| 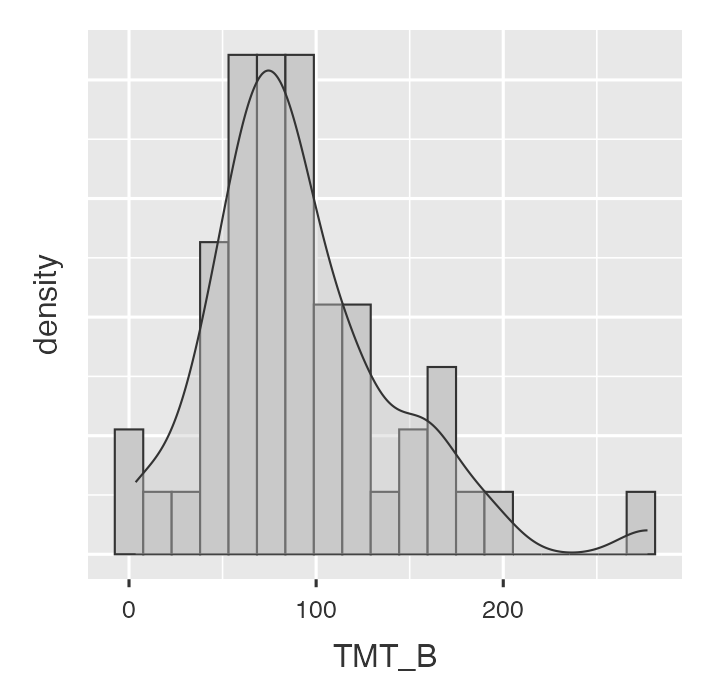 | 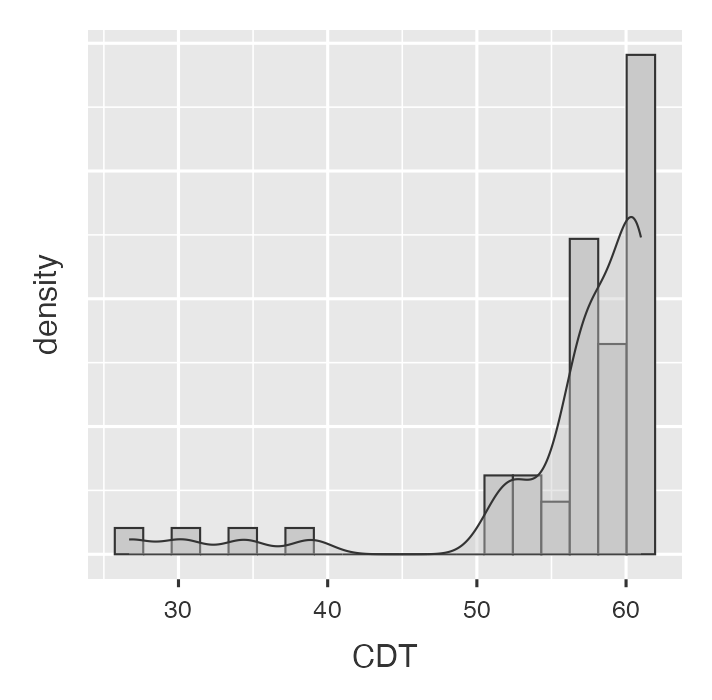 | 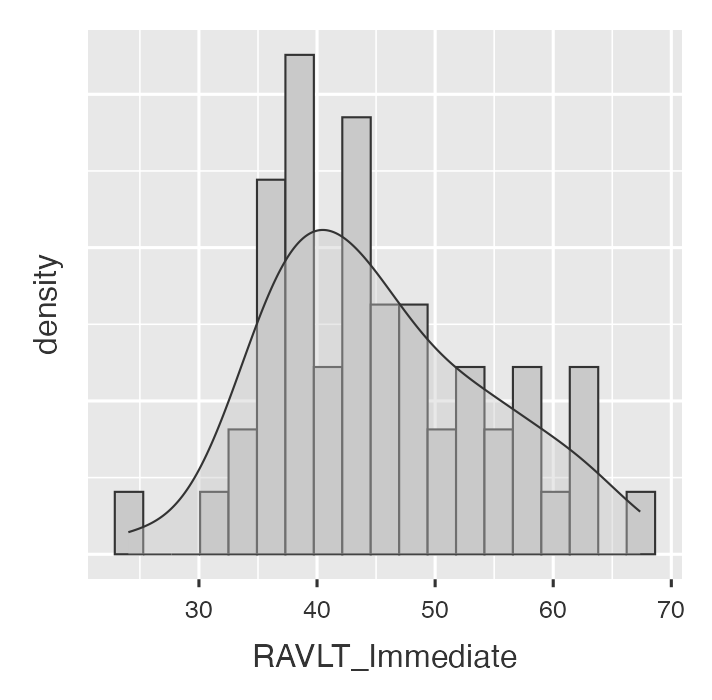 |
| 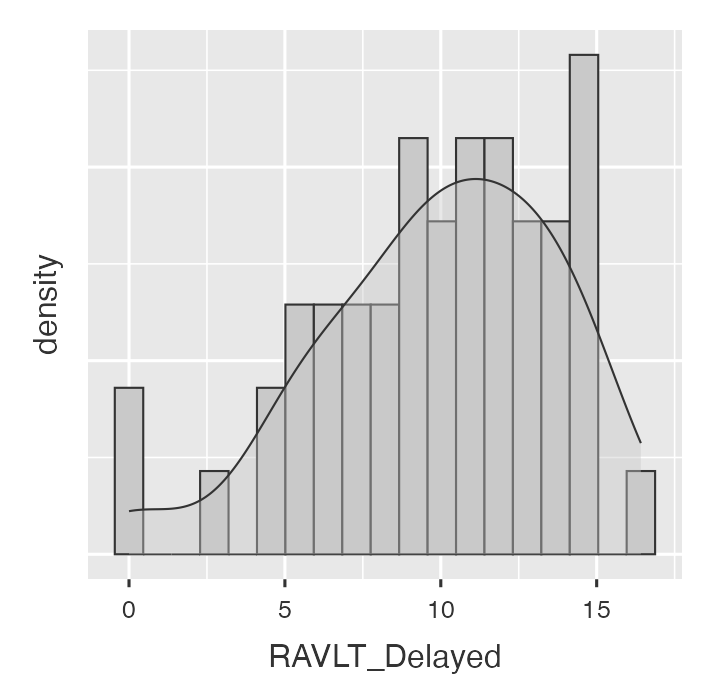 | 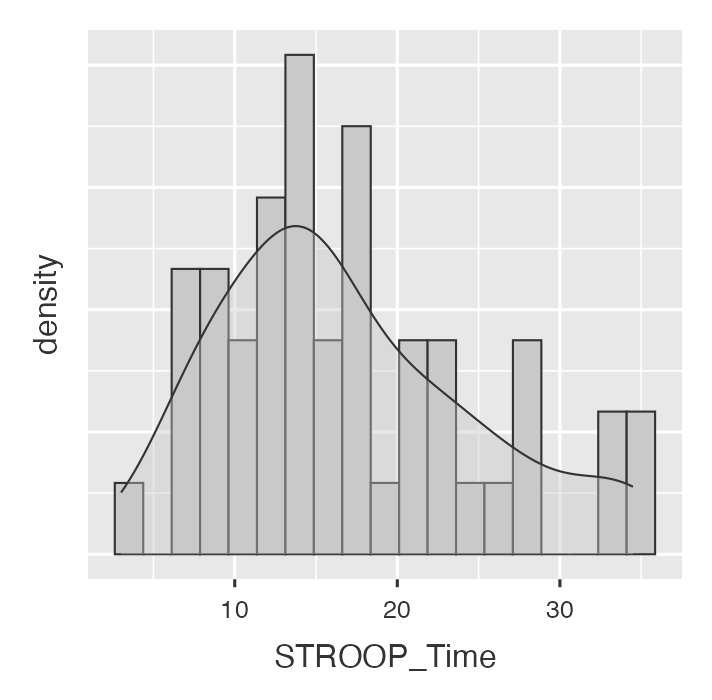 | 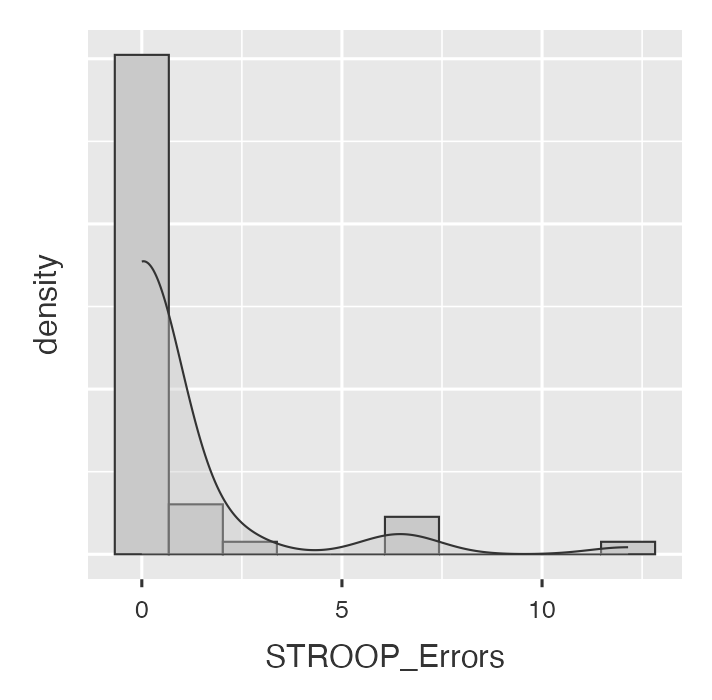 |

| **Descriptives psychologial variables** | | | | | | | | | | | |
| --- | --- | --- | --- | --- | --- | --- | --- | --- | --- | --- | --- |
|  | | | | | | **Skewness** | | **Kurtosis** | | **Shapiro-Wilk** | |
|  | **N** | **Missing** | **Mean** | **Median** | **SD** | **Skewness** | **SE** | **Kurtosis** | **SE** | **W** | **p** |
| **CFI_self_report** | 41 | 10 | 4.70 | 4.50 | 2.71 | 0.392 | 0.369 | -0.455 | 0.724 | 0.961 | 0.170 |
| **GAD_7** | 51 | 0 | 6.10 | 5 | 4.33 | 1.128 | 0.333 | 0.596 | 0.656 | 0.882 | <.001 |
| **PHQ_9** | 51 | 0 | 7.16 | 6 | 5.23 | 1.025 | 0.333 | 1.049 | 0.656 | 0.923 | 0.003 |
| **MASCoD** | 49 | 2 | 11.14 | 11 | 3.56 | -0.301 | 0.340 | -0.442 | 0.668 | 0.969 | 0.211 |

| 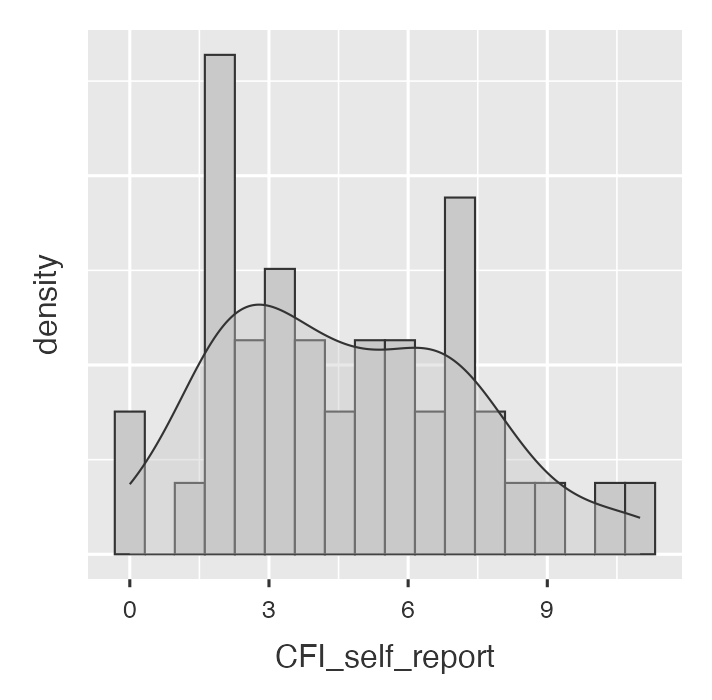 | 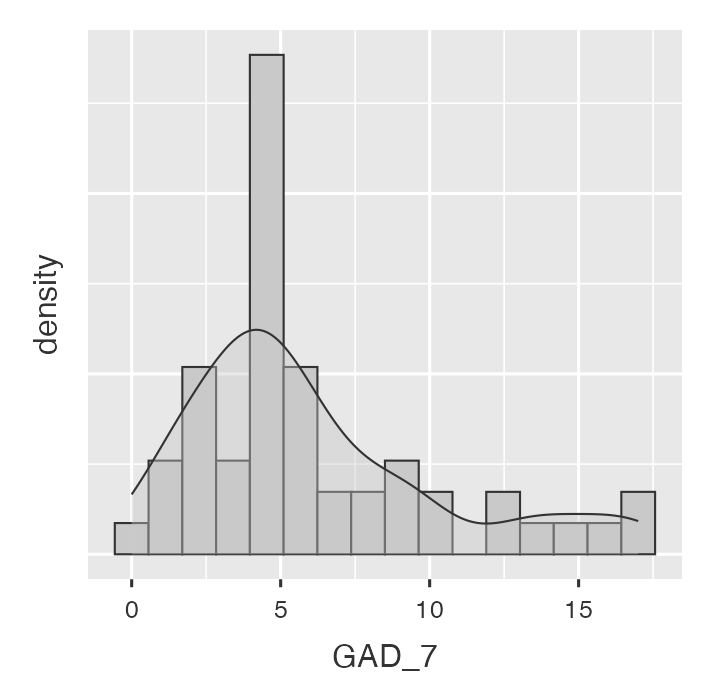 |
| --- | --- |
| 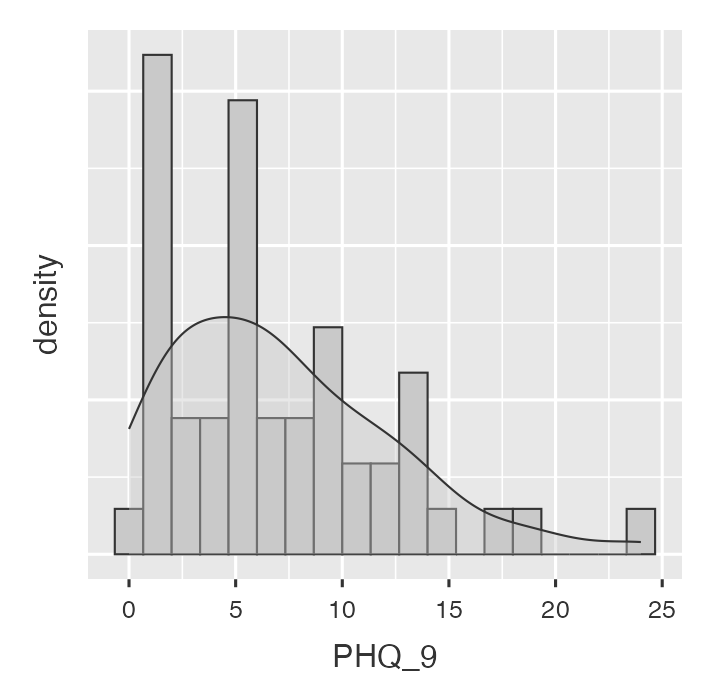 | 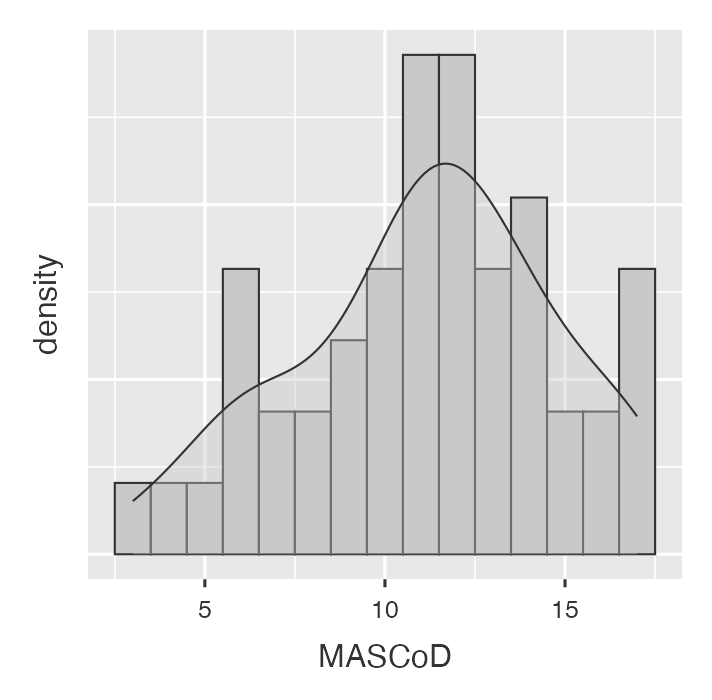 |

| **Descriptives of cognitive reserve** | | | | | | | | | | | |
| --- | --- | --- | --- | --- | --- | --- | --- | --- | --- | --- | --- |
|  | | | | | | **Skewness** | | **Kurtosis** | | **Shapiro-Wilk** | |
|  | **N** | **Missing** | **Mean** | **Median** | **SD** | **Skewness** | **SE** | **Kurtosis** | **SE** | **W** | **p** |
| **CRIq_total** | 51 | 0 | 105 | 101 | 16.4 | 0.640 | 0.333 | -0.249 | 0.656 | 0.955 | 0.051 |
| **CRIq_Education** | 51 | 0 | 103 | 101 | 11.2 | 0.539 | 0.333 | -0.717 | 0.656 | 0.942 | 0.015 |
| **CRIq_Working_activity** | 51 | 0 | 101 | 95 | 19.5 | 0.747 | 0.333 | -0.297 | 0.656 | 0.932 | 0.006 |
| **CRIq_Leisure_Time** | 51 | 0 | 108 | 103 | 24.7 | 0.600 | 0.333 | -0.205 | 0.656 | 0.961 | 0.089 |

| 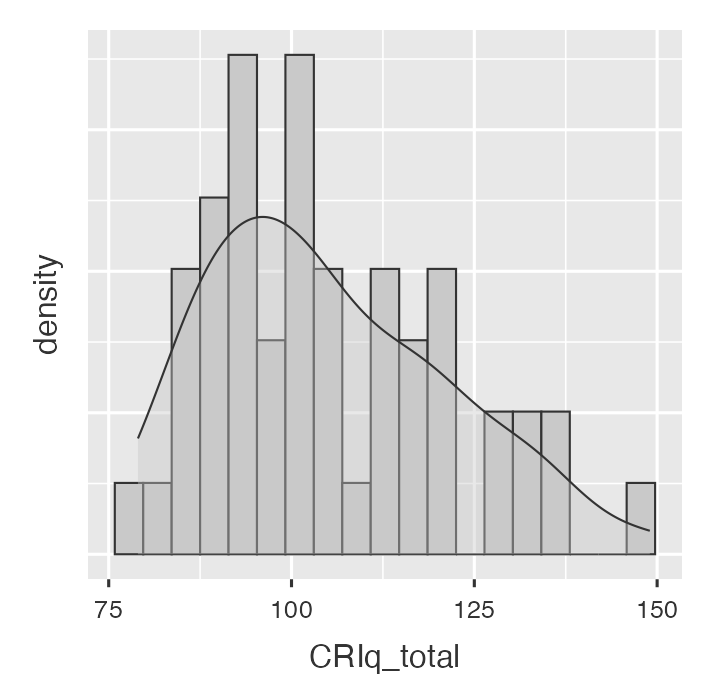 | 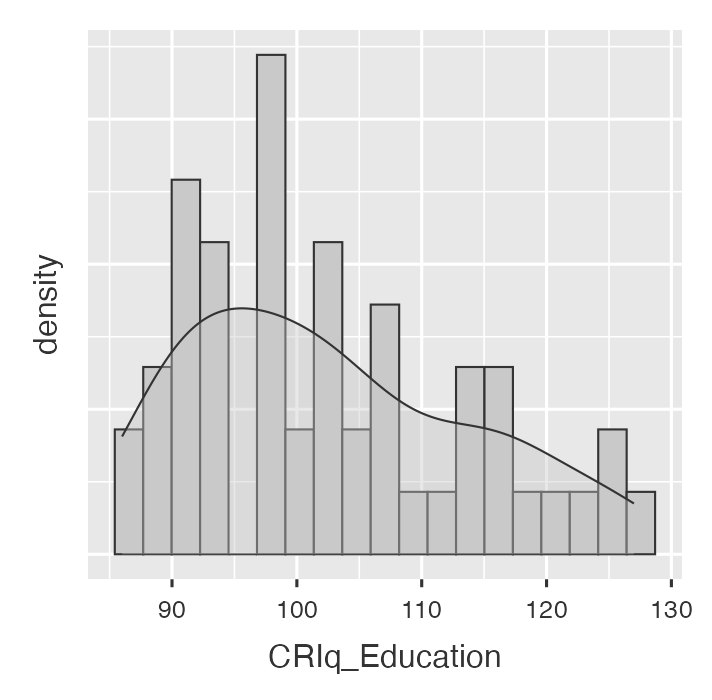 |
| --- | --- |
| 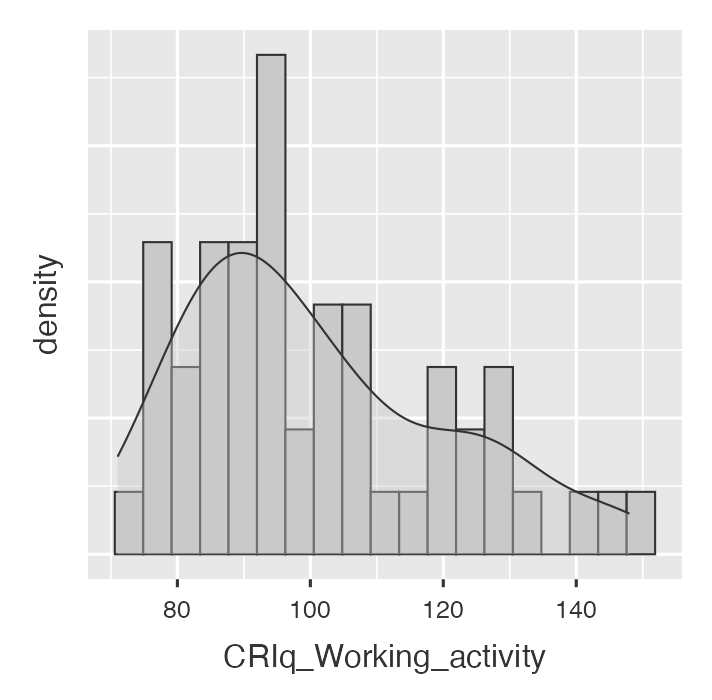 | 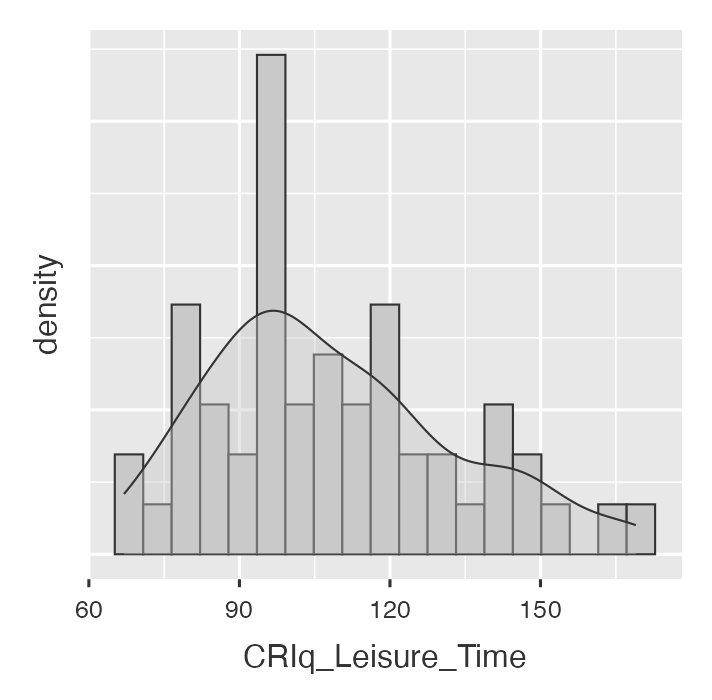 |

**Kruskal–Wallis comparisons of demographic and risk factors across cognitive reserve groups based (low, medium, high) on the CRIq Total.**

|  | **X^2^** | **df** | **p** |
| --- | --- | --- | --- |
| **Kruskal-Wallis** |  |  |  |
| Sex | 0.889 | 2 | 0.641 |
| Age | 2.611 | 2 | 0.271 |
| Physical Activity | 3.116 | 2 | 0.211 |
| Smoking | 0.394 | 2 | 0.821 |
| Family History of NCDs | 0.813 | 2 | 0.666 |
| Dyslipidemia | 2.353 | 2 | 0.308 |
| Hypertension | 1.189 | 2 | 0.552 |
| Diabetes | 7.016 | 2 | 0.030 |
| Overweight | 0.482 | 2 | 0.786 |
| Alcohol | 0.989 | 2 | 0.610 |

|  |  | **W** | **p** |
| --- | --- | --- | --- |
| **DSCF– Diabetes** | | | |
| High | Medium | 3.05 | 0.079 |
| High | Low | 3.84 | 0.018 |
| Medium | Low | 1.03 | 0.745 |

**Kruskal–Wallis comparisons of demographic and risk factors across cognitive reserve groups based (low, medium, high) on the CRIq Education.**

|  | **X^2^** | **df** | **p** |
| --- | --- | --- | --- |
| **Kruskal-Wallis** |  |  |  |
| Sex | 0.278 | 2 | 0.870 |
| Age | 0.294 | 2 | 0.863 |
| Physical Activity | 6.929 | 2 | 0.031 |
| Smoking | 2.702 | 2 | 0.259 |
| Family History of NCDs | 3.081 | 2 | 0.214 |
| Dyslipidemia | 1.364 | 2 | 0.506 |
| Hypertension | 1.919 | 2 | 0.383 |
| Diabetes | 1.001 | 2 | 0.606 |
| Overweight | 0.284 | 2 | 0.868 |
| Alcohol | 0.516 | 2 | 0.772 |

|  |  | **W** | **p** |
| --- | --- | --- | --- |
| **DSCF– Physical Activity** | | | |
| High | Medium | 3.541 | 0.033 |
| High | Low | 2.863 | 0.106 |
| Medium | Low | -0.599 | 0.906 |

**Kruskal–Wallis comparisons of demographic and risk factors across cognitive reserve groups based (low, medium, high) on the CRIq Working Activity.**

|  | **X^2^** | **df** | **p** |
| --- | --- | --- | --- |
| **Kruskal-Wallis** |  |  |  |
| Sex | 5.5729 | 2 | 0.062 |
| Age | 2.9519 | 2 | 0.229 |
| Physical Activity | 2.1419 | 2 | 0.343 |
| Smoking | 4.5450 | 2 | 0.103 |
| Family History of NCDs | 2.6451 | 2 | 0.266 |
| Dyslipidemia | 0.9935 | 2 | 0.609 |
| Hypertension | 0.0453 | 2 | 0.978 |
| Diabetes | 1.1052 | 2 | 0.575 |
| Overweight | 0.0385 | 2 | 0.981 |
| Alcohol | 1.3992 | 2 | 0.497 |

**Kruskal–Wallis comparisons of demographic and risk factors across cognitive reserve groups based (low, medium, high) on the CRIq Leisure Time.**

|  | **X^2^** | **df** | **p** |
| --- | --- | --- | --- |
| **Kruskal-Wallis** |  |  |  |
| Sex | 0.2068 | 2 | 0.902 |
| Age | 0.0917 | 2 | 0.955 |
| Physical Activity | 1.3018 | 2 | 0.522 |
| Smoking | 0.7725 | 2 | 0.680 |
| Family History of NCDs | 2.5416 | 2 | 0.281 |
| Dyslipidemia | 0.7928 | 2 | 0.673 |
| Hypertension | 3.6612 | 2 | 0.160 |
| Diabetes | 4.5176 | 2 | 0.104 |
| Overweight | 0.4821 | 2 | 0.786 |
| Alcohol | 0.0199 | 2 | 0.990 |

**Spearman correlations**

| Correlation Matrix | | | | | | | |
| --- | --- | --- | --- | --- | --- | --- | --- |
|  |  | **CRIq_total** | **CRIq_Education** | **CRIq_Working_activity** | **CRIq_Leisure_Time** | **CFI_self_report** | **MASCoD** |
| **CRIq_total** | **Spearman's rho** | — |  |  |  |  |  |
|  | **df** | — |  |  |  |  |  |
|  | **p-value** | — |  |  |  |  |  |
| **CRIq_Education** | **Spearman's rho** | 0.523*** | — |  |  |  |  |
|  | **df** | 49 | — |  |  |  |  |
|  | **p-value** | <.001 | — |  |  |  |  |
| **CRIq_Working_activity** | **Spearman's rho** | 0.719*** | 0.460*** | — |  |  |  |
|  | **df** | 49 | 49 | — |  |  |  |
|  | **p-value** | <.001 | <.001 | — |  |  |  |
| **CRIq_Leisure_Time** | **Spearman's rho** | 0.708*** | 0.070 | 0.114 | — |  |  |
|  | **df** | 49 | 49 | 49 | — |  |  |
|  | **p-value** | <.001 | 0.625 | 0.425 | — |  |  |
| **CFI_self_report** | **Spearman's rho** | -0.064 | 0.028 | -0.200 | 0.029 | — |  |
|  | **df** | 39 | 39 | 39 | 39 | — |  |
|  | **p-value** | 0.689 | 0.864 | 0.211 | 0.855 | — |  |
| **MASCoD** | **Spearman's rho** | 0.059 | 0.114 | -0.048 | 0.045 | 0.409** | — |
|  | **df** | 47 | 47 | 47 | 47 | 37 | — |
|  | **p-value** | 0.688 | 0.437 | 0.741 | 0.759 | 0.010 | — |
| Note. * p < .05, ** p < .01, *** p < .001 | | | | | | | |

**Kruskal–Wallis comparisons of global cognitive functioning across cognitive reserve groups based (low, medium, high) on the CRI Total.**

|  | **X^2^** | **df** | **p** | **ε²** | **Rank** | **Critical value** | **p_FDR** |
| --- | --- | --- | --- | --- | --- | --- | --- |
| **Kruskal-Wallis** |  |  |  |  |  |  |  |
| MMSE | 5.44 | 2 | 0.066 | 0.109 | 1 | 0.025 | 0.104 |
| ACE-III | 4.53 | 2 | 0.104 | 0.105 | 2 | 0.05 | 0.104 |

**Kruskal–Wallis comparisons of global cognitive functioning across cognitive reserve groups based (low, medium, high) on the CRI Education.**

|  | **X^2^** | **df** | **p** | **ε²** | **Rank** | **Critical value** | **p_FDR** |
| --- | --- | --- | --- | --- | --- | --- | --- |
| **Kruskal-Wallis** |  |  |  |  |  |  |  |
| MMSE | 1.20 | 2 | 0.549 | 0.0240 | 2 | 0.05 | 0.549 |
| ACE-III | 3.72 | 2 | 0.156 | 0.0864 | 1 | 0.025 | 0.312 |

**Kruskal–Wallis comparisons of global cognitive functioning across cognitive reserve groups based (low, medium, high) on the CRI Working Activivity.**

|  | **X^2^** | **df** | **p** | **ε²** | **Rank** | **Critical value** | **p_FDR** |
| --- | --- | --- | --- | --- | --- | --- | --- |
| **Kruskal-Wallis** |  |  |  |  |  |  |  |
| MMSE | 4.05 | 2 | 0.132 | 0.0810 | 1 | 0.025 | 0.264 |
| ACE-III | 2.32 | 2 | 0.313 | 0.0540 | 2 | 0.05 | 0.313 |

**Kruskal–Wallis comparisons of global cognitive functioning across cognitive reserve groups based (low, medium, high) on the CRI Leisure Time.**

|  | **X^2^** | **df** | **p** | **ε²** | **Rank** | **Critical value** | **p_FDR** |
| --- | --- | --- | --- | --- | --- | --- | --- |
| **Kruskal-Wallis** |  |  |  |  |  |  |  |
| MMSE | 1.875 | 2 | 0.392 | 0.0375 | 1 | 0.025 | 0.676 |
| ACE-III | 0.784 | 2 | 0.676 | 0.0182 | 2 | 0.05 | 0.676 |

**Kruskal–Wallis comparisons of attention and executive functioning across cognitive reserve groups based (low, medium, high) on the CRI Total.**

|  | **X^2^** | **df** | **p** | **ε²** | **Rank** | **Critical value** | **p_FDR** |
| --- | --- | --- | --- | --- | --- | --- | --- |
| **Kruskal-Wallis** |  |  |  |  |  |  |  |
| FAB | 0.908 | 2 | 0.635 | 0.01815 | 4 | 0.04 | 0.79375 |
| TMT-A | 2.512 | 2 | 0.285 | 0.05024 | 3 | 0.03 | 0.475 |
| TMT-B | 3.839 | 2 | 0.147 | 0.07834 | 2 | 0.02 | 0.3675 |
| STROOP Time | 0.459 | 2 | 0.795 | 0.00957 | 5 | 0.05 | 0.795 |
| STROOP Errors | 8.910 | 2 | 0.012 | 0.18562 | 1 | 0.01 | 0.06 |

**Kruskal–Wallis comparisons of attention and executive functioning across cognitive reserve groups based (low, medium, high) on the CRI Education.**

|  | **X^2^** | **df** | **p** | **ε²** | **Rank** | **Critical value** | **p_FDR** |
| --- | --- | --- | --- | --- | --- | --- | --- |
| **Kruskal-Wallis** |  |  |  |  |  |  |  |
| FAB | 0.201 | 2 | 0.904 | 0.00402 | 5 | 0.05 | 0.9040 |
| TMT-A | 3.604 | 2 | 0.165 | 0.07208 | 2 | 0.02 | 0.26 |
| TMT-B | 3.137 | 2 | 0.208 | 0.06401 | 3 | 0.03 | 0.26 |
| STROOP Time | 5.090 | 2 | 0.078 | 0.10605 | 1 | 0.01 | 0.26 |
| STROOP Errors | 3.143 | 2 | 0.208 | 0.06547 | 4 | 0.04 | 0.26 |

**Kruskal–Wallis comparisons of attention and executive functioning across cognitive reserve groups based (low, medium, high) on the CRI Working Activity.**

|  | **X^2^** | **df** | **p** | **ε²** | **Rank** | **Critical value** | **p_FDR** |
| --- | --- | --- | --- | --- | --- | --- | --- |
| **Kruskal-Wallis** |  |  |  |  |  |  |  |
| FAB | 1.953 | 2 | 0.377 | 0.0391 | 2 | 0.02 | 0.5863 |
| TMT-A | 0.520 | 2 | 0.771 | 0.0104 | 5 | 0.05 | 0.771 |
| TMT-B | 1.514 | 2 | 0.469 | 0.0309 | 4 | 0.04 | 0.5863 |
| STROOP Time | 1.785 | 2 | 0.410 | 0.0372 | 3 | 0.03 | 0.5863 |
| STROOP Errors | 7.028 | 2 | 0.030 | 0.1464 | 1 | 0.01 | 0.15 |

**Kruskal–Wallis comparisons of attention and executive functioning across cognitive reserve groups based (low, medium, high) on the CRI Leisure Time.**

|  | **X^2^** | **df** | **p** | **ε²** | **Rank** | **Critical value** | **p_FDR** |
| --- | --- | --- | --- | --- | --- | --- | --- |
| **Kruskal-Wallis** |  |  |  |  |  |  |  |
| FAB | 1.79 | 2 | 0.410 | 0.0357 | 5 | 0.05 | 0.410 |
| TMT-A | 9.69 | 2 | 0.008 | 0.1938 | 2 | 0.02 | 0.02 |
| TMT-B | 10.36 | 2 | 0.006 | 0.2115 | 1 | 0.01 | 0.02 |
| STROOP Time | 2.44 | 2 | 0.295 | 0.0508 | 4 | 0.04 | 0.36875 |
| STROOP Errors | 6.09 | 2 | 0.048 | 0.1269 | 3 | 0.03 | 0.08 |

|  |  | **W** | **p** |
| --- | --- | --- | --- |
| **DSCF– TMT-A** | | | |
| Low | Medium | 4.14 | 0.010 |
| Low | High | 3.32 | 0.049 |
| Medium | High | -1.16 | 0.689 |

|  |  | **W** | **p** |
| --- | --- | --- | --- |
| **DSCF– TMT-B** | | | |
| Low | Medium | 4.032 | 0.012 |
| Low | High | 3.886 | 0.017 |
| Medium | High | -0.467 | 0.942 |

**Kruskal–Wallis comparisons of language across cognitive reserve groups based (low, medium, high) on the CRI Total.**

|  | **X^2^** | **df** | **p** | **ε²** | **Rank** | **Critical value** | **p_FDR** |
| --- | --- | --- | --- | --- | --- | --- | --- |
| **Kruskal-Wallis** |  |  |  |  |  |  |  |
| Phonemic | 2.53 | 2 | 0.282 | 0.0506 | 1 | 2 | 0.05 |
| Semantic | 6.07 | 2 | 0.048 | 0.1239 | 2 | 1 | 0.025 |

**Kruskal–Wallis comparisons of language across cognitive reserve groups based (low, medium, high) on the CRI Education.**

|  | **X^2^** | **df** | **p** | **ε²** | **Rank** | **Critical value** | **p_FDR** |
| --- | --- | --- | --- | --- | --- | --- | --- |
| **Kruskal-Wallis** |  |  |  |  |  |  |  |
| Phonemic | 1.26 | 2 | 0.533 | 0.0252 | 2 | 0.05 | 0.533 |
| Semantic | 4.90 | 2 | 0.086 | 0.1000 | 1 | 0.025 | 0.172 |

**Kruskal–Wallis comparisons of language across cognitive reserve groups based (low, medium, high) on the CRI Working Activity.**

|  | **X^2^** | **df** | **p** | **ε²** | **Rank** | **Critical value** | **p_FDR** |
| --- | --- | --- | --- | --- | --- | --- | --- |
| **Kruskal-Wallis** |  |  |  |  |  |  |  |
| Phonemic | 2.28 | 2 | 0.319 | 0.0457 | 2 | 0.05 | 0.319 |
| Semantic | 2.30 | 2 | 0.316 | 0.0470 | 1 | 0.025 | 0.319 |

**Kruskal–Wallis comparisons of language across cognitive reserve groups based (low, medium, high) on the CRI Leisure Time.**

|  | **X^2^** | **df** | **p** | **ε²** | **Rank** | **Critical value** | **p_FDR** |
| --- | --- | --- | --- | --- | --- | --- | --- |
| **Kruskal-Wallis** |  |  |  |  |  |  |  |
| Phonemic | 0.0591 | 2 | 0.971 | 0.00118 | 2 | 0.05 | 0.971 |
| Semantic | 0.2015 | 2 | 0.904 | 0.00411 | 1 | 0.025 | 0.971 |

**Kruskal–Wallis comparisons of short-term memory across cognitive reserve groups based (low, medium, high) on the CRI Total.**

|  | **X^2^** | **df** | **p** | **ε²** | **Rank** | **Critical value** | **p_FDR** |
| --- | --- | --- | --- | --- | --- | --- | --- |
| **Kruskal-Wallis** |  |  |  |  |  |  |  |
| DSF | 1.071 | 2 | 0.585 | 0.02142 | 1 | 0.025 | 0.866 |
| CSF | 0.289 | 2 | 0.866 | 0.00578 | 2 | 0.05 | 0.866 |

**Kruskal–Wallis comparisons of short-term memory across cognitive reserve groups based (low, medium, high) on the CRI Education.**

|  | **X^2^** | **df** | **p** | **ε²** | **Rank** | **Critical value** | **p_FDR** |
| --- | --- | --- | --- | --- | --- | --- | --- |
| **Kruskal-Wallis** |  |  |  |  |  |  |  |
| DSF | 0.389 | 2 | 0.823 | 0.00778 | 2 | 0.05 | 0.823 |
| CSF | 0.417 | 2 | 0.812 | 0.00835 | 1 | 0.025 | 0.823 |

**Kruskal–Wallis comparisons of short-term memory across cognitive reserve groups based (low, medium, high) on the CRI Working Activity.**

|  | **X^2^** | **df** | **p** | **ε²** | **Rank** | **Critical value** | **p_FDR** |
| --- | --- | --- | --- | --- | --- | --- | --- |
| **Kruskal-Wallis** |  |  |  |  |  |  |  |
| DSF | 7.22 | 2 | 0.027 | 0.1445 | 1 | 0.025 | 0.054 |
| CSF | 1.59 | 2 | 0.452 | 0.0318 | 2 | 0.05 | 0.452 |

**Kruskal–Wallis comparisons of short-term memory across cognitive reserve groups based (low, medium, high) on the CRI Leisure Time.**

|  | **X^2^** | **df** | **p** | **ε²** | **Rank** | **Critical value** | **p_FDR** |
| --- | --- | --- | --- | --- | --- | --- | --- |
| **Kruskal-Wallis** |  |  |  |  |  |  |  |
| DSF | 3.14 | 2 | 0.208 | 0.0628 | 1 | 0.025 | 0.416 |
| CSF | 1.02 | 2 | 0.601 | 0.0204 | 2 | 0.05 | 0.601 |

**Kruskal–Wallis comparisons of working memory across cognitive reserve groups based (low, medium, high) on the CRI Total.**

|  | **X^2^** | **df** | **p** | **ε²** | **Rank** | **Critical value** | **p_FDR** |
| --- | --- | --- | --- | --- | --- | --- | --- |
| **Kruskal-Wallis** |  |  |  |  |  |  |  |
| DSB | 0.519 | 2 | 0.772 | 0.0104 | 2 | 0.05 | 0.772 |
| CSB | 1.522 | 2 | 0.467 | 0.0304 | 1 | 0.025 | 0.772 |

**Kruskal–Wallis comparisons of working memory across cognitive reserve groups based (low, medium, high) on the CRI Education.**

|  | **X^2^** | **df** | **p** | **ε²** | **Rank** | **Critical value** | **p_FDR** |
| --- | --- | --- | --- | --- | --- | --- | --- |
| **Kruskal-Wallis** |  |  |  |  |  |  |  |
| DSB | 0.913 | 2 | 0.633 | 0.0183 | 2 | 0.05 | 0.633 |
| CSB | 0.971 | 2 | 0.615 | 0.0194 | 1 | 0.025 | 0.633 |

**Kruskal–Wallis comparisons of working memory across cognitive reserve groups based (low, medium, high) on the CRI Working Activity.**

|  | **X^2^** | **df** | **p** | **ε²** | **Rank** | **Critical value** | **p_FDR** |
| --- | --- | --- | --- | --- | --- | --- | --- |
| **Kruskal-Wallis** |  |  |  |  |  |  |  |
| DSB | 0.3929 | 2 | 0.822 | 0.00786 | 1 | 0.025 | 0.992 |
| CSB | 0.0157 | 2 | 0.992 | 3.13e-4 | 2 | 0.05 | 0.992 |

**Kruskal–Wallis comparisons of working memory across cognitive reserve groups based (low, medium, high) on the CRI Leisure Time.**

|  | **X^2^** | **df** | **p** | **ε²** | **Rank** | **Critical value** | **p_FDR** |
| --- | --- | --- | --- | --- | --- | --- | --- |
| **Kruskal-Wallis** |  |  |  |  |  |  |  |
| DSB | 4.086 | 2 | 0.130 | 0.0817 | 1 | 0.025 | 0.26 |
| CSB | 0.832 | 2 | 0.660 | 0.0166 | 2 | 0.05 | 0.660 |

**Kruskal–Wallis comparisons of long-term memory across cognitive reserve groups based (low, medium, high) on the CRI Total.**

|  | **X^2^** | **df** | **p** | **ε²** | **Rank** | **Critical value** | **p_FDR** |
| --- | --- | --- | --- | --- | --- | --- | --- |
| **Kruskal-Wallis** |  |  |  |  |  |  |  |
| ROCF delayed | 1.793 | 2 | 0.408 | 0.03586 | 2 | 0,033 | 0.612 |
| RAVLT immediate | 0.225 | 2 | 0.894 | 0.00450 | 3 | 0,05 | 0.894 |
| RAVLT delayed | 2.024 | 2 | 0.364 | 0.04047 | 1 | 0,0167 | 0.612 |

**Kruskal–Wallis comparisons of long-term memory across cognitive reserve groups based (low, medium, high) on the CRI Education.**

|  | **X^2^** | **df** | **p** | **ε²** | **Rank** | **Critical value** | **p_FDR** |
| --- | --- | --- | --- | --- | --- | --- | --- |
| **Kruskal-Wallis** |  |  |  |  |  |  |  |
| ROCF delayed | 2.37 | 2 | 0.305 | 0.0474 | 1 | 0.0167 | 0.56 |
| RAVLT immediate | 1.16 | 2 | 0.560 | 0.0232 | 3 | 0.05 | 0.56 |
| RAVLT delayed | 1.86 | 2 | 0.395 | 0.0372 | 2 | 0.033 | 0.56 |

**Kruskal–Wallis comparisons of long-term memory across cognitive reserve groups based (low, medium, high) on the CRI Working Activity.**

|  | **X^2^** | **df** | **p** | **ε²** | **Rank** | **Critical value** | **p_FDR** |
| --- | --- | --- | --- | --- | --- | --- | --- |
| **Kruskal-Wallis** |  |  |  |  |  |  |  |
| ROCF delayed | 0.745 | 2 | 0.689 | 0.0149 | 3 | 0.05 | 0.689 |
| RAVLT immediate | 3.571 | 2 | 0.168 | 0.0714 | 2 | 0.033 | 0.252 |
| RAVLT delayed | 5.659 | 2 | 0.059 | 0.1132 | 1 | 0.0167 | 0.177 |

**Kruskal–Wallis comparisons of long-term memory across cognitive reserve groups based (low, medium, high) on the CRI Leisure Time.**

|  | **X^2^** | **df** | **p** | **ε²** | **Rank** | **Critical value** | **p_FDR** |
| --- | --- | --- | --- | --- | --- | --- | --- |
| **Kruskal-Wallis** |  |  |  |  |  |  |  |
| ROCF delayed | 0.273 | 2 | 0.872 | 0.00546 | 3 | 0.05 | 0.872 |
| RAVLT immediate | 4.321 | 2 | 0.115 | 0.08642 | 1 | 0.0167 | 0.345 |
| RAVLT delayed | 1.713 | 2 | 0.425 | 0.03427 | 2 | 0.033 | 0.6375 |

**Kruskal–Wallis comparisons of constructive praxis and visuospatial abilities across cognitive reserve groups based (low, medium, high) on the CRI Total.**

|  | **X^2^** | **df** | **p** | **ε²** | **Rank** | **Critical value** | **p_FDR** |
| --- | --- | --- | --- | --- | --- | --- | --- |
| **Kruskal-Wallis** |  |  |  |  |  |  |  |
| ROCF copy | 7.48 | 2 | 0.024 | 0.1496 | 1 | 0.025 | 0.048 |
| CDT | 1.55 | 2 | 0.462 | 0.0309 | 2 | 0.05 | 0.462 |

|  |  | **W** | **p** |
| --- | --- | --- | --- |
| **DSCF– ROCF copy** | | | |
| Low | Medium | 3.250 | 0.056 |
| Low | High | 3.505 | 0.035 |
| Medium | High | -0.336 | 0.969 |

**Kruskal–Wallis comparisons of constructive praxis and visuospatial abilities across cognitive reserve groups based (low, medium, high) on the CRI Education.**

|  | **X^2^** | **df** | **p** | **ε²** | **Rank** | **Critical value** | **p_FDR** |
| --- | --- | --- | --- | --- | --- | --- | --- |
| **Kruskal-Wallis** |  |  |  |  |  |  |  |
| ROCF copy | 0.660 | 2 | 0.719 | 0.0132 | 2 | 0.05 | 0.719 |
| CDT | 6.723 | 2 | 0.035 | 0.1345 | 1 | 0.025 | 0.07 |

**Kruskal–Wallis comparisons of constructive praxis and visuospatial abilities across cognitive reserve groups based (low, medium, high) on the CRI Working Activity.**

|  | **X^2^** | **df** | **p** | **ε²** | **Rank** | **Critical value** | **p_FDR** |
| --- | --- | --- | --- | --- | --- | --- | --- |
| **Kruskal-Wallis** |  |  |  |  |  |  |  |
| ROCF copy | 6.48 | 2 | 0.039 | 0.1296 | 1 | 0.025 | 0.078 |
| CDT | 3.73 | 2 | 0.155 | 0.0747 | 2 | 0.05 | 0.155 |

**Kruskal–Wallis comparisons of constructive praxis and visuospatial abilities across cognitive reserve groups based (low, medium, high) on the CRI Leisure Time.**

|  | **X^2^** | **df** | **p** | **ε²** | **Rank** | **Critical value** | **p_FDR** |
| --- | --- | --- | --- | --- | --- | --- | --- |
| **Kruskal-Wallis** |  |  |  |  |  |  |  |
| ROCF copy | 4.61 | 2 | 0.100 | 0.0922 | 1 | 0.025 | 0.200 |
| CDT | 1.12 | 2 | 0.573 | 0.0223 | 2 | 0.05 | 0.573 |

**Kruskal–Wallis comparisons of psychological conditions across cognitive reserve groups based (low, medium, high) on the CRI Total.**

|  | **X^2^** | **df** | **p** | **ε²** | **Rank** | **Critical value** | **p_FDR** |
| --- | --- | --- | --- | --- | --- | --- | --- |
| **Kruskal-Wallis** |  |  |  |  |  |  |  |
| PHQ-9 | 2.75 | 2 | 0.253 | 0.0550 | 2 | 0.025 | 0.44 |
| GAD-7 | 2.11 | 2 | 0.349 | 0.0421 | 3 | 0.0375 | 0.44 |
| CFI | 4.33 | 2 | 0.115 | 0.1083 | 1 | 0.0125 | 0.44 |
| MASCoD | 1.64 | 2 | 0.440 | 0.0342 | 4 | 0.05 | 0.44 |

**Kruskal–Wallis comparisons of psychological conditions across cognitive reserve groups based (low, medium, high) on the CRI Education.**

|  | **X^2^** | **df** | **p** | **ε²** | **Rank** | **Critical value** | **p_FDR** |
| --- | --- | --- | --- | --- | --- | --- | --- |
| **Kruskal-Wallis** |  |  |  |  |  |  |  |
| PHQ-9 | 0.287 | 2 | 0.866 | 0.00574 | 4 | 0.05 | 0.866 |
| GAD-7 | 4.443 | 2 | 0.108 | 0.08885 | 1 | 0.0125 | 0.432 |
| CFI | 0.971 | 2 | 0.616 | 0.02426 | 2 | 0.025 | 0.866 |
| MASCoD | 0.430 | 2 | 0.806 | 0.00896 | 3 | 0.0375 | 0.866 |

**Kruskal–Wallis comparisons of psychological conditions across cognitive reserve groups based (low, medium, high) on the CRI Working Activity.**

|  | **X^2^** | **df** | **p** | **ε²** | **Rank** | **Critical value** | **p_FDR** |
| --- | --- | --- | --- | --- | --- | --- | --- |
| **Kruskal-Wallis** |  |  |  |  |  |  |  |
| PHQ-9 | 9.19 | 2 | 0.010 | 0.1838 | 1 | 0.0125 | 0.0400 |
| GAD-7 | 4.59 | 2 | 0.101 | 0.0918 | 2 | 0.025 | 0.202 |
| CFI | 1.44 | 2 | 0.486 | 0.0361 | 3 | 0.0375 | 0.542 |
| MASCoD | 1.23 | 2 | 0.542 | 0.0255 | 4 | 0.05 | 0.542 |

|  |  | **W** | **p** |
| --- | --- | --- | --- |
| **DSCF– PHQ-9** | | | |
| Low | Medium | -3.86 | 0.017 |
| Low | High | -1.08 | 0.726 |
| Medium | High | 3.43 | 0.041 |

**Kruskal–Wallis comparisons of psychological conditions across cognitive reserve groups based (low, medium, high) on the CRI Leisure Time.**

|  | **X^2^** | **df** | **p** | **ε²** | **Rank** | **Critical value** | **p_FDR** |
| --- | --- | --- | --- | --- | --- | --- | --- |
| **Kruskal-Wallis** |  |  |  |  |  |  |  |
| PHQ-9 | 0.0833 | 2 | 0.959 | 0.00167 | 2 | 0,025 | 0.972 |
| GAD-7 | 0.0571 | 2 | 0.972 | 0.00114 | 4 | 0,05 | 0.972 |
| CFI | 1.9546 | 2 | 0.376 | 0.04887 | 1 | 0,0125 | 0.972 |
| MASCoD | 0.0838 | 2 | 0.959 | 0.00175 | 3 | 0.0375 | 0.972 |
